# Different aspects of Alzheimer’s disease-related amyloid β-peptide pathology and their relationship to amyloid positron emission tomography imaging and dementia

**DOI:** 10.1186/s40478-019-0837-9

**Published:** 2019-11-14

**Authors:** Dietmar Rudolf Thal, Alicja Ronisz, Thomas Tousseyn, Ajeet Rijal Upadhaya, Karthikeyan Balakrishnan, Rik Vandenberghe, Mathieu Vandenbulcke, Christine A. F. von Arnim, Markus Otto, Thomas G. Beach, Johan Lilja, Kerstin Heurling, Aruna Chakrabarty, Azzam Ismail, Christopher Buckley, Adrian P. L. Smith, Sathish Kumar, Gill Farrar, Jochen Walter

**Affiliations:** 1 Department of Imaging and Pathology, KU-Leuven, Leuven, Belgium; 2 Department of Pathology, UZ-Leuven, Leuven, Belgium; 3 Leuven Brain Institute, KU-Leuven, Leuven, Belgium; 4 Laboratory for Neuropathology – Institute of Pathology, University of Ulm, Ulm, Germany; 5 Department of Gene Therapy, University of Ulm, Ulm, Germany; 6 Department of Neurosciences, KU-Leuven, Herestraat 49, 3000 Leuven, Belgium; 7 Department of Neurology, UZ-Leuven, Leuven, Belgium; 8 Department of Geriatric Psychiatry, UZ-Leuven, Leuven, Belgium; 9 Department of Neurology, University of Ulm, Ulm, Germany; 10 Department of Geriatrics, University Medical Center Göttingen, Göttingen, Germany; 11 Civin Laboratory for Neuropathology, Banner Sun Health Research Institute, Sun City, AZ USA; 12 Hermes Medical Solutions AB, Stockholm, Sweden; 13 Department of Psychiatry and Neurochemistry, Wallenberg Centre for Molecular and Translational Medicine, University of Gothenburg, Gothenborg, Sweden; 14 Pathology and Tumour Biology, Leeds Institute of Molecular Medicine, St. James Hospital, Leeds, UK; 15 GE Healthcare Life Sciences, Amersham, UK; 16 Department of Neurology, University of Bonn, Bonn, Germany

**Keywords:** Alzheimer’s disease, Amyloid β peptide, Staging, Amyloid load, Soluble amyloid, Insoluble amyloid, Amyloid maturation, Amyloid PET, [^18^F]flutemetamol

## Abstract

Alzheimer’s disease (AD)-related amyloid β-peptide (Aβ) pathology in the form of amyloid plaques and cerebral amyloid angiopathy (CAA) spreads in its topographical distribution, increases in quantity, and undergoes qualitative changes in its composition of modified Aβ species throughout the pathogenesis of AD. It is not clear which of these aspects of Aβ pathology contribute to AD progression and to what extent amyloid positron emission tomography (PET) reflects each of these aspects. To address these questions three cohorts of human autopsy cases (in total *n* = 271) were neuropathologically and biochemically examined for the topographical distribution of Aβ pathology (plaques and CAA), its quantity and its composition. These parameters were compared with neurofibrillary tangle (NFT) and neuritic plaque pathology, the degree of dementia and the results from [^18^F]flutemetamol amyloid PET imaging in cohort 3. All three aspects of Aβ pathology correlated with one another, the estimation of Aβ pathology by [^18^F]flutemetamol PET, AD-related NFT pathology, neuritic plaques, and with the degree of dementia. These results show that one aspect of Aβ pathology can be used to predict the other two, and correlates well with the development of dementia, advancing NFT and neuritic plaque pathology. Moreover, amyloid PET estimates all three aspects of Aβ pathology in-vivo. Accordingly, amyloid PET-based estimates for staging of amyloid pathology indicate the progression status of amyloid pathology in general and, in doing so, also of AD pathology. Only 7.75% of our cases deviated from this general association.

## Introduction

The deposition of amyloid β-peptide (Aβ) in amyloid plaques is one of the histopathological hallmark lesions of Alzheimer’s disease (AD) [47] together with neurofibrillary tangles (NFTs) [35]. Neuritic plaques represent a subset of the Aβ plaques characterized by the presence of dystrophic neurites in the plaques that can be stained with antibodies against abnormal τ-protein [11, 18, 35, 51, 70]. In addition to its presence in amyloid plaques, Aβ can also be found in cerebral and leptomeningeal blood vessels affected by cerebral amyloid angiopathy (CAA) [25], as soluble, dispersible Aβ in extra- or intracellular fluid, and as membrane-associated Aβ aggregates [14, 29, 56, 58, 81]. Aβ pathology can be described by 1. the topographical distribution of Aβ plaques [11, 71, 72] or CAA-affected vessels in the brain [68], 2. measures of quantity in a given brain region (morphological Aβ plaque loads, biochemically detected measures of Aβ levels (ELISA, western blotting)) [2, 3, 14, 41, 46, 50, 57, 59, 81], and 3. qualitative changes in the composition of detectable modified and non-modified Aβ species, such as Aβ_40/42_, Aβ_N3pE_ and Aβ_pSer8_ [4, 24, 38, 44, 57, 61, 75]. Aβ aggregation, thereby, starts with the accumulation of amyloid fibrils that are only detectable with antibodies detecting non-modified forms, followed by the presence of Aβ_N3pE_ and finally by Aβ_pSer8_, indicating a process called “maturation of Aβ aggregates” [75]. However, it is not yet clear to what extent a) the different aspects of Aβ pathology, i.e. the topographical distribution of plaques and CAA-affected vessels, the quantitative and qualitative measures of Aβ aggregation and deposition, correlate with one another, b) predict the development of dementia and c) can be estimated in-vivo by amyloid PET.

To address these questions, three cohorts of autopsy cases were neuropathologically analyzed for the topographical distribution of Aβ plaques and CAA. These parameters were correlated with the clinical degree of dementia and neuropathological measures for NFT and neuritic plaque pathology in all three cohorts, quantitative measures for plaque loads in two cohorts, biochemically detected levels of Aβ in one cohort, the morphologically and biochemically determined stages of Aβ aggregates maturation in one cohort, and with the [2] flutemetamol PET estimates for the phase of Aβ plaque deposition in another cohort.

## Material and methods

### Subjects

The findings of 3 cohorts of autopsy cases (Table 1) were combined where possible and reassessed. The first cohort of 95 cases represents autopsy cases from university and municipal hospitals in Bonn, Ulm and Offenbach am Main (Germany) [57, 71, 73], a second novel cohort of 79 cases represents cases from the university hospital in Leuven (Belgium), and the third cohort of 97 cases was included in the efficacy analysis of the [^18^F] flutemetamol phase III autopsy study (ClinicalTrials.gov identifiers NCT01165554, and NCT02090855) [9, 36]. Local institutional review boards or ethics committees approved the study protocols before initiation [17, 66]. This study covering samples from three cohorts was approved by the UZ/KU-Leuven ethical committee (S-59295).

**Table 1:** Age, gender distribution of the 3 patient cohorts and the respective distribution of Aβ phases, Aβ-MTL phases, A-scores, Braak NFT-stage, CERAD neuritic plaque score, NIA-AA degree of AD pathology, diagnosis, PET-Aβ phase estimate, B-Aβ stage, B-Aβ plaque stage, Aβ load. Short description of the recruitment criteria of the cohorts and case selection criteria for this study

	Cohort 1	Cohort 2	Cohort 3
	German cases	Leuven cases	[18F]flutemetamol phase 3 autopsy cases
Number of cases	95	79	97
Age in years (mean/range)	72,43 (35–98)	67,43 (34–90)	80,86 (59–95)
male/female	48/47	54/25	45/52
Aβ phase (mean/range)	2,34 (0–5)	1,8 (0–5)	3,65 (0–5)
AβMTL phase (mean/range)	1,96 (0–4)	1,47 (0–5)	2,92 (0–4)
A-score (mean/range)	1,49 (0–3)	1,18 (0–3)	2,39 (0–3)
Braak NFT-stage (mean/range)	2,11 (0–6)	2,27 (0–6)	3,95 (0–6)
CERAD neuritic plaque score (mean/range)	0,53 (0–3)	0,66 (0–3)	1,86 (0–3)
NIA-AA degree of AD pathology (mean/range)	1,11 (0–3)	0,97 (0–3)	2,01 (0–3)
Diagnosis (control/p-preAD/AD/AD+non-AD-D*/non-AD-D*)	24/35/13/5/18	18/4/15/8/34	3/8/33/28/25
PET-Aβ phase estimate (mean/range)	n.a.	n.a.	1,81 (0–3)
Aβ load / % (mean/range)	4,16 (0–23,34)	n.a.	6,75 (0–17,63)
B-Aβ stage (mean/range)	1,42 (0–3) [*n* = 38]	n.a.	n.a.
B-Aβ plaque stage (mean/range)	1,74 (0–3) [*n* = 70]	n.a.	n.a.
CAA severity grade (Vonsattel)	1 (0–3)	0,84 (0–3)	1,51 (0–3)
CAA stage of topographical distribution	1,1 (0–3)	0,72 (0–3)	1,64 (0–3)
CDR	0,993 (0–3) [*n* = 88]	1622 (0–3) [*n* = 74]	n.a.
MMSE	n.a.	n.a.	9,48 (0–30) [*n* = 65]
Scan-death interval	n.a.	n.a.	215 (0–846) days
Recruitment strategy	Hospital-based autopsies	Memory clinic-based cohort	Terminally ill with life-expectancy of less than 3 years, ≥55 years of age, no pregnancy, no allergy against [^18^F]flutemetamol, physical status allows to undergo PET imaging
Case selection criteria	Aβ phases, AβMTL phases and Aβ loads already determined in the context of previous studies	Aβ phases and AβMTL phases already determined for biobank purposes	[^18^F]flutemetamol amyloid PET images are available that allow the measurement of SUVRcort and SUVRcaud

^*^Non-AD dementia (non-AD-D) includes cases with Lewy body disease, frontotemporal lobar degeneration with TDP43 (FTLD-TDP), fused in sarcoma (FTLD-FUS), or τ pathology (FTLD-tau: progressive supranuclear palsy, corticobasal degeneration, argyrophilic grain disease, Pick’s disease, and neurofibrillary tangle predominant dementia), encephalitis, Creutzfeldt-Jacob disease, tumor, vascular dementia and metabolic encephalopathy. These non-AD-D cases served as non-AD control cases from diseased brains to determine the differential diagnostic properties of the respective parameters

The non-AD and pathologically diagnosed preclinical cases with AD pathology (p-preAD) [57] of cohorts 1 and 2 were examined at the time point of hospital admission (approximately 1 to 4 weeks prior to death) by clinicians with different specialties according to standardized protocols. The AD patients were diagnosed by a neurologist (RV, MV, MO, CAFvA) and followed until death. The protocols included the assessment of cognitive function (orientation to place, time and person; specific cognitive or neuropsychiatric tests were not performed) and recorded the patients’ ability to care for themselves and to get dressed, eating habits, bladder and bowel continence, speech patterns, writing and reading ability, short-term and long-term memory, and orientation within the hospital setting. These data were used to retrospectively assess the clinical dementia rating (CDR) scores for 162/174 patients [34] without knowledge of the pathological diagnosis. For this purpose, the information from the clinical files was used to provide a CDR score according to the standard CDR protocol [34]. For 12 cases of cohorts 1 and 2 the clinical data were not sufficient to obtain a CDR score retrospectively. In cohort 3, the clinical data provided mini-mental state examination (MMSE) [23] test results from 65 cases.

### Neuropathology

Autopsy was performed with informed consent of the patients during life or the next in kin after death of the patient. From cohorts 1–3 one brain hemisphere was cut fresh and specimens were kept frozen for biochemical analysis. The other hemisphere was fixed in a 4% aqueous solution of formaldehyde (cohort 1) or phosphate buffered formaldehyde (cohorts 2–3) for ca. 3–4 weeks before cutting. In the event that tissue was considered as suspicious for Creutzfeldt-Jakob disease no frozen tissue was collected and the formalin-fixed tissue was decontaminated in 99% formic acid. Brains were cut in coronal slices and screened macroscopically. For histopathological analysis and for assessing the amounts of AD-related amyloid plaques, NFTs, and neuritic plaques, paraffin-embedded tissue including parts of the frontal, parietal, temporal, occipital cortex, and entorhinal cortex, the hippocampal formation at the level of the lateral geniculate body, basal ganglia, thalamus, amygdala, midbrain, pons, medulla oblongata and cerebellum were examined. Paraffin sections of 5–12 μm thickness from all blocks were stained with hematoxylin & eosin. For neuropathological diagnosis sections were stained with the Gallyas (cohort 1) or Bielschowsky (cohort 3) silver method and immunohistochemical methods (cohorts 1–3) with primary antibodies against abnormal phosphorylated τ protein (p-τ), Aβ_17–24_, Aβ_N3pE_, Aβ_pSer8_ [40], phosphorylated transactive DNA-binding protein TDP43 (pTDP43), α-synuclein, and/or ubiquitin as listed in Additional file 1: Table S1. Primary antibodies were detected with biotinylated secondary antibodies and visualized with the DABMap Kit (Ventana, USA) or with diaminobencidine-HCl and the avidin-biotin complex (Vector, USA).

The phase of Aβ plaque pathology (Aβ phase) was assessed after screening the Aβ-stained sections for plaque distribution according to previously published protocols (Additional file 1: Table S2). One single amyloid plaque, thereby, indicated that a given anatomical brain region was considered as amyloid positive [1, 71]. The neuropathological diagnosis of AD pathology as well as the assessment of the A-score (A0 – A3) for amyloid plaque distribution and the determination of the NIA-AA degree of AD pathology was performed as recommended [35] (Additional file 1: Table S2). Braak-NFT staging was performed based on sections stained with an antibody against abnormal τ-protein (AT8, Additional file 1: Table S1) according to a widely accepted protocol in all cohorts [10]. In some cohort 1 cases, Braak NFT-staging was confirmed with the Gallyas silver staining method [11]. The consortium to establish a registry for AD (CERAD) scores for neuritic plaque density were assessed based on sections stained with an antibody against abnormal τ-protein (AT8, Additional file 1: Table S1) [51].

As an additional staging strategy for the topographical distribution of Aβ plaques we used the phases of Aβ plaque distribution in the medial temporal lobe (AβMTL phases) [72]. The assessment of the Aβ phases, A-scores, and AβMTL phases was carried out according to the protocol depicted in Additional file 1: Table S2a-d. In addition, the severity of CAA according to Vonsattel [80], and the stage of topographical expansion of CAA have been assessed as previously described [68] (for details see Additional file 1: Table S2e,f).

To assess the quantitative aspect of Aβ pathology, Aβ-loads were determined in all cases of cohort 1 and in 31 cases of cohort 3 as the percentage of the area in the temporal neocortex (Brodmann area 36) covered by Aβ plaques detected with anti-Aβ_17–24_. Morphometry for Aβ load determination in cohort 1 was performed using ImageJ image processing and analysis software (National Institutes of Health, Bethesda, USA). For plaque measurements, the area of the morphologically identified plaques was interactively delineated with a cursor and then measured. Neuronal staining by the anti-Aβ_17–24_ antibody was considered as cross-reactivity with amyloid precursor protein (APP) and not included for the assessment of the Aβ load. The areas of all plaques in a given cortical region were added up. The area of the respective cortex areas was likewise measured by interactive delineation with a cursor. The Aβ load was calculated as the percentage of the area of interest covered by Aβ plaques [56]. Likewise, Aβ_N3pE_ and Aβ_pSer8_ loads were assessed in 70 cases of cohort 1 cases. In cohort 3 the cortical Aβ loads were assessed in the middle temporal gyrus after scanning the 4G8-stained sections with a slide scanner and performing automated analysis of cortical regions of interest with Aperio XT software and a pre-developed macro (MATLAB, Math Work In, MA, USA) [36]. Thresholds for intensity, size, and morphometry were set by the macro to distinguish Aβ plaques from non-plaque related neuronal APP staining after color deconvolution to remove the hematoxylin staining channel. These Aβ load measures for cohort 3 were performed at a single laboratory to ensure consistency. The investigators were blinded to clinical and imaging data and to the results of other histopathology analyses.

To document qualitative changes in the aggregate composition, the stage of maturation of Aβ plaques (B-Aβ plaque stage) was determined in 70 cases of cohort 1 (Tab. 1) [57]. To do so, sections were immunostained with antibodies raised against Aβ_17–24_, Aβ_N3pE_, and Aβ_pSer8_. Four B-Aβ plaques stages were distinguished (for details see Additional file 1: Table S3a).

### Biochemistry

Biochemical analysis was carried out from 38 cases of cohort 1 (Tab. 1) [57]. Protein extraction from fresh frozen occipital and temporal neocortex (0.4 g) was carried out in 2 ml of 0.32 M sucrose dissolved in Tris-buffered saline (TBS) containing a protease and phosphatase inhibitor-cocktail (Complete and PhosSTOP, Roche, Mannheim, Germany). The tissue was first homogenized with Micropestle (Eppendorf, Hamburg, Germany) followed by sonication (Sonoplus HD 2070, Heidolph instruments, Germany). The homogenate was centrifuged for 30 min at 14000 x *g* at 4 °C. The supernatant (s1) was ultracentrifuged to at 175000 x *g* to separate the soluble fraction (supernatant s2) and the dispersible fraction (pellet p2). The pellet (p2) resuspended in TBS and the supernatant as the soluble fraction (s2) were stored at − 80 °C until further use, respectively. The pellet (p1) containing the membrane-associated and the solid plaque-associated fraction was resuspended in TBS containing 2% sodium dodecyl sulfate (SDS) was centrifuged at 14000 x *g*. The supernatant (s3) was kept as membrane-associated SDS soluble fraction. The pellet (p3) was further dissolved in 70% formic acid and the homogenate was lyophilized by centrifuging in the vacuum centrifuge (Vacufuge; Eppendorf, Germany) and reconstituted in 100 μl of 2X lithium dodecyl sulfate (LDS) sample buffer (Invitrogen, Carlsbad, CA, USA) followed by heating at 70 °C for 5 min. The resultant sample was considered as plaque-associated, formic acid-soluble fraction [48]. The total protein contents of soluble, dispersible, and membrane-associated fractions were determined using BCA Protein Assay (Bio-Rad, Hercules, CA, USA).

For western blot analysis, the four fractions (soluble, dispersible, membrane-associated and plaque-associated) were subjected to SDS-polyacrylamide gel electrophoresis (SDS-PAGE) and subsequent western blot analysis with anti-Aβ_1–17_, anti-Aβ_N3pE_ and anti-Aβ_pSer8_ antibodies (Additional file 1: Table S1). Blots were developed with an ECL detection system (Supersignal Pico Western system, ThermoScientific-Pierce, Waltham, MA, USA) and illuminated in ECL Hyperfilm (GE Healthcare, Buckinghamshire, UK). For semiquantitative comparison of optical densities of the 4 kDa Aβ bands were measured using ImageJ software (NIH, Bethesda, USA) as previously described [56].

The biochemical stages of Aβ aggregation (B-Aβ stages) were determined by the detection of the presence/absence of Aβ, Aβ_N3pE_, and Aβ_pSer8_ in at least one of the four fractions according to a previously published protocol (for detail see Additional file 1: Table S3b) [57].

### [^18^F]Flutemetamol PET image assessments

Amyloid PET imaging was performed for the cases in cohort 3 at 12 different imaging sites [62, 66]. Before PET imaging, subjects underwent head CT or magnetic resonance imaging (MRI), unless prior images (obtained within 12 months) were available. [^18^F]Flutemetamol injection was administered intravenously at a dose of 185 or 370 MBq of radioactivity at physician discretion [66]. PET images were acquired in 2.5-min frames on PET/CT cameras, beginning approximately 90 min post injection, which was attenuation corrected using CT data. Frame to frame motion correction was performed on the dynamic data before the frames were averaged to give a 10-min scan. Equipment used to capture images varied across the 12 imaging sites [66]. Most images were reconstructed iteratively to form 128 × 128 axial slices, and a Gaussian post-reconstruction smoothing filter was applied to some to achieve uniform image resolution across sites.

[^18^F]flutemetamol uptake was measured for six volumes of interest (VOIs) restricted to gray matter and adjusted for atrophy manually where possible, covering anterior cingulate, prefrontal cortex, lateral temporal cortex, parietal cortex, one VOI covering both posterior cingulate and precuneus, and one subcortical VOI in the head of the caudate nucleus according to Thal et al. [67] and Beach et al. [9]. Quantitative standardized uptake value ratio (SUVR) calculations were made using pons as reference region [76]. A global cortical average (neocortical (composite) SUVR (SUVRneo); obtained from anterior cingulate, prefrontal, lateral temporal, parietal, and posterior cingulate cortex including the precuneus region) was calculated [79]. The SUVR for the caput nuclei caudati (SUVRcaud) was determined based on VOI measurements of both the left and right caudate nucleus (anterior aspect). The caudate VOIs were drawn on a parasagittal plane which intersected the thalamus, internal capsule, caudate head and frontal white matter (manually due to the lack of structural MRI for most cases). Image processing and VOI analysis was performed using VOIager 4.0.7 (GE Healthcare, Uppsala, Sweden) [67].

Thresholds to distinguish Aβ phases by [^18^F] flutemetamol PET based estimates were applied as recently published (for detail see Additional file 1: Table S4) [67].

### Statistical analysis

Spearman correlation, partial correlation, linear regression analysis and regression coefficients were calculated using SPSS 25 statistical software (IBM, Armonk, NY, USA). To exclude collinearity with age and sex when comparing Aβ-related parameters with non-Aβ-related parameters partial correlation and regression analyses were controlled for age and sex. For comparisons of the different aspects of Aβ pathology with one another the Spearman correlation analysis was used without controlling for age and sex in order not to bias these comparisons of different aspects of aggregates of the same molecule by including additional independent variables in the respective model terms. The Friedman test was used to compare Aβ, Aβ_N3pE_, and Aβ_pSer8_ in dependent samples to observe differences in the respective levels and loads. Pairwise comparisons were adjusted for multiple testing according to the Bonferroni method.

## Results

The main findings of this study were (1) strong correlations between the topographical (Aβ phases, AβMTL phases, A-scores, CAA stages, and CAA-severity), quantitative (Aβ loads, Aβ levels determined biochemically in cortical brain homogenates), and qualitative (B-Aβ stages for Aβ aggregate/Aβ plaque maturation) aspects of Aβ pathology, (2) estimation of these aspects by the SUVR-based PET-Aβ phase estimates, and (3) its relationship with preclinical and symptomatic stages of AD and cognitive decline.

### Correlations between topographical, quantitative and qualitative aspects of Aβ pathology

Spearman correlation analysis was applied to determine correlations between topographical (Aβ phases, AβMTL phases, A-scores, CAA stage, and CAA severity), quantitative (Aβ, Aβ_N3pE_, and Aβ_pSer8_ loads, biochemically detected levels of soluble, dispersible, membrane-associated and plaque-associated Aβ, Aβ_N3pE_, and Aβ_pSer8_), and qualitative Aβ parameters (B-Aβ stages, and B-Aβ plaque stages) in cohort 1. This analysis revealed strong correlations between these parameters (Additional file 1: Table S5a). The Aβ_pSer8_ levels in the plaque-associated fraction did not correlate with the B-Aβ plaque stage (*p* = 0.094; Additional file 1: Table S5a). Soluble Aβ_pSer8_ was not detectable. The correlations between topographical parameters (Aβ phases, AβMTL phases, A-scores, CAA stage, and CAA severity) were confirmed in cohorts 2 and 3 (Additional file 1: Table S5b). The correlation of topographical parameters with Aβ load was confirmed in cohort 3 (Additional file 1: Table S5c).

**Fig. 1:**
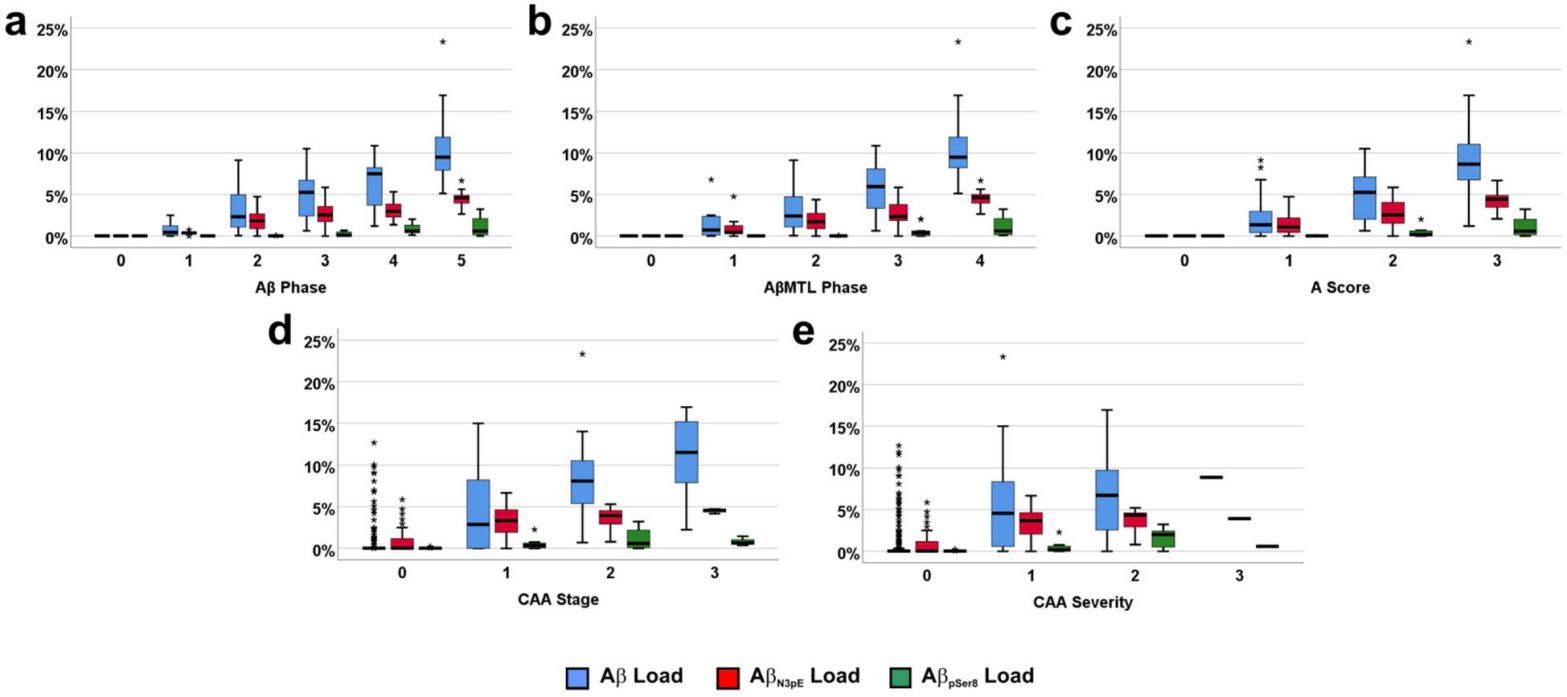
Comparison of the relationship of the Aβ phases (**a**), AβMTL phases (**b**), the A-scores (**c**), CAA stages (**d**) and CAA severities (**e**) with Aβ plaque loads for Aβ, Aβ_N3pE_, and Aβ_pSer8_-containig plaques in cohort 1, depicted by boxplot diagrams. **a**: The plaque-loads for Aβ (Spearman correlation analysis: *r* = 0.888) and Aβ_N3pE_ (Spearman correlation analysis: *r* = 0.882) correlated better with the Aβ phases than that for Aβ_pSer8_-positive plaques (Spearman correlation analysis: *r* = 0.810). **b-e**: Likewise, the AβMTL phases (**b**), the A-scores (**c**), the CAA stage (**d**) and the CAA severity (**e**) correlated with the Aβ, Aβ_N3pE_, and Aβ_pSer8_ loads (*r* = 0.582–0.899; *p* < 0.001; for detailed statistical analysis see Additional file 1: Table S5a)

In detail, with increasing Aβ phase, AβMTL phase, and A-score, the Aβ load increased showing in general higher levels than the Aβ_N3pE_ load and the Aβ_pSer8_ load (Fig. 1a-c; Friedman test corrected for multiple testing: *p* = 0.001). Increased Aβ_pSer8_ loads were mainly restricted to end stages (Aβ phases 4 and 5, AβMTL phase 4, and A-score 3) and lower than Aβ_N3pE_ loads (Fig. 1a-c; Friedman test corrected for multiple testing: *p* < 0.001). Cases without CAA but with Aβ and Aβ_N3pE_ loads larger than 0 were seen with Aβ loads ranging from 0.02 to 9.78% (cohort 1)/ 0.017 to 17.625% (cohort 3) with a mean of 3.068% (cohort 1)/ 7.17% (cohort 3) (Fig. 1d, e). In general, Aβ, Aβ_N3pE_, and Aβ_pSer8_ loads increased with advancing topographical expansion of CAA pathology over the brain (represented by the CAA stage), and with increasing destruction of the vessel wall of cortical and leptomeningeal blood vessels by Aβ deposits (represented by the CAA severity) (Fig. 1d, e). Aβ_pSer8_ was thereby, seen in cases with CAA stage 2 and CAA severity degree 2.

**Fig. 2:**
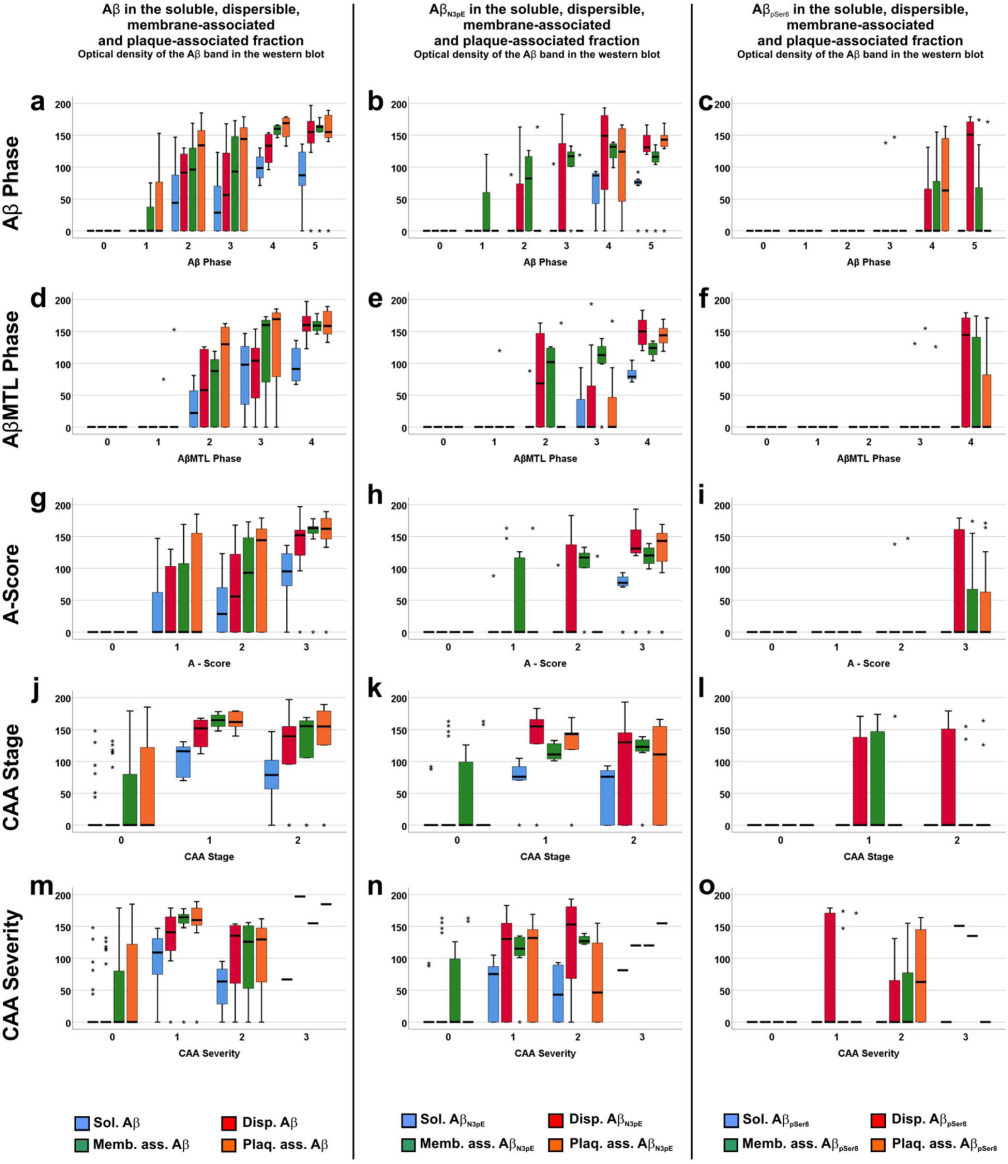
Boxplot diagrams representing the distribution of soluble (Sol.), dispersible (Disp.), membrane-associated (Memb. ass.), and plaque-associated (Plaq. Ass.) Aβ (**a, d, g, j, m**), Aβ_N3pE_ (**b, e, h, k, n**), and Aβ_pSer8_ levels (**c, f, i, l, o**) in relation to the phase of Aβ plaque distribution (Aβ phase; **a-c**), the AβMTL phase (**d-f**), the A-score (**g-i**), the CAA stages (**j-l**) and the CAA severity (**m**-**o**) in cohort 1. The correlation for these three different forms of Aβ was best for Aβ detected with antibodies against non-modified forms of Aβ (Spearman correlation analysis: *r* = 0.603–0.809) followed by Aβ_N3pE_ (Spearman correlation analysis: *r* = 0.572–0.756) whereas Aβ_pSer8_ was not detectable in soluble Aβ aggregates. Dispersible, membrane-associated and plaque-associated Aβ_pSer8_ showed a week correlation with the Aβ phases (Spearman correlation analysis: *r* = 0.324–0.556) due to the fact that it was seen only in Aβ phases 4 and 5 but not earlier, except for single cases. Increased levels of Aβ and Aβ_N3pE_ were found already in cases without CAA (**m, n**). Cases with CAA showed high levels of soluble, dispersible, membrane-associated, and plaque-associated Aβ and Aβ_N3pE_ in all stage and severity degrees of CAA. Only the presence of Aβ_pSer8_ was restricted to cases with CAA. The detailed correlation analysis is provided in Additional file 1: Table S5a

A similar correlation relation was observed for the biochemically detected levels of soluble, membrane-associated and plaque-associated Aβ, Aβ_N3pE_, and Aβ_pSer8_ increasing with the five topographical parameters for Aβ pathology, except for soluble Aβ_pSer8_ (Fig. 2), which was not detected in these cases. The levels of soluble, dispersible, membrane-associated, and plaque-associated total Aβ were higher than that of the respective levels of Aβ_pSer8_ (Fig. 2; Friedman test corrected for multiple testing: *p* < 0.001–0.025). Eight cases without CAA showed Aβ and Aβ_N3pE_ mainly in the membrane-associated and the plaque-associated fraction. The levels of Aβ, Aβ_N3pE_, and Aβ_pSer8_ increased in CAA stage 1 and remained similar in stage 2. Biochemical data from CAA stage 3 cases were not available for this analysis. A similar pattern was observed for the severity degree of CAA. There was an increase until CAA severity degree 1 (mild CAA). In CAA severity degree 2 and 3 cases, similar levels of soluble, dispersible, membrane-associated and plaque-associated Aβ, Aβ_N3pE_, and Aβ_pSer8_ were observed (Fig. 2).

**Fig. 3:**
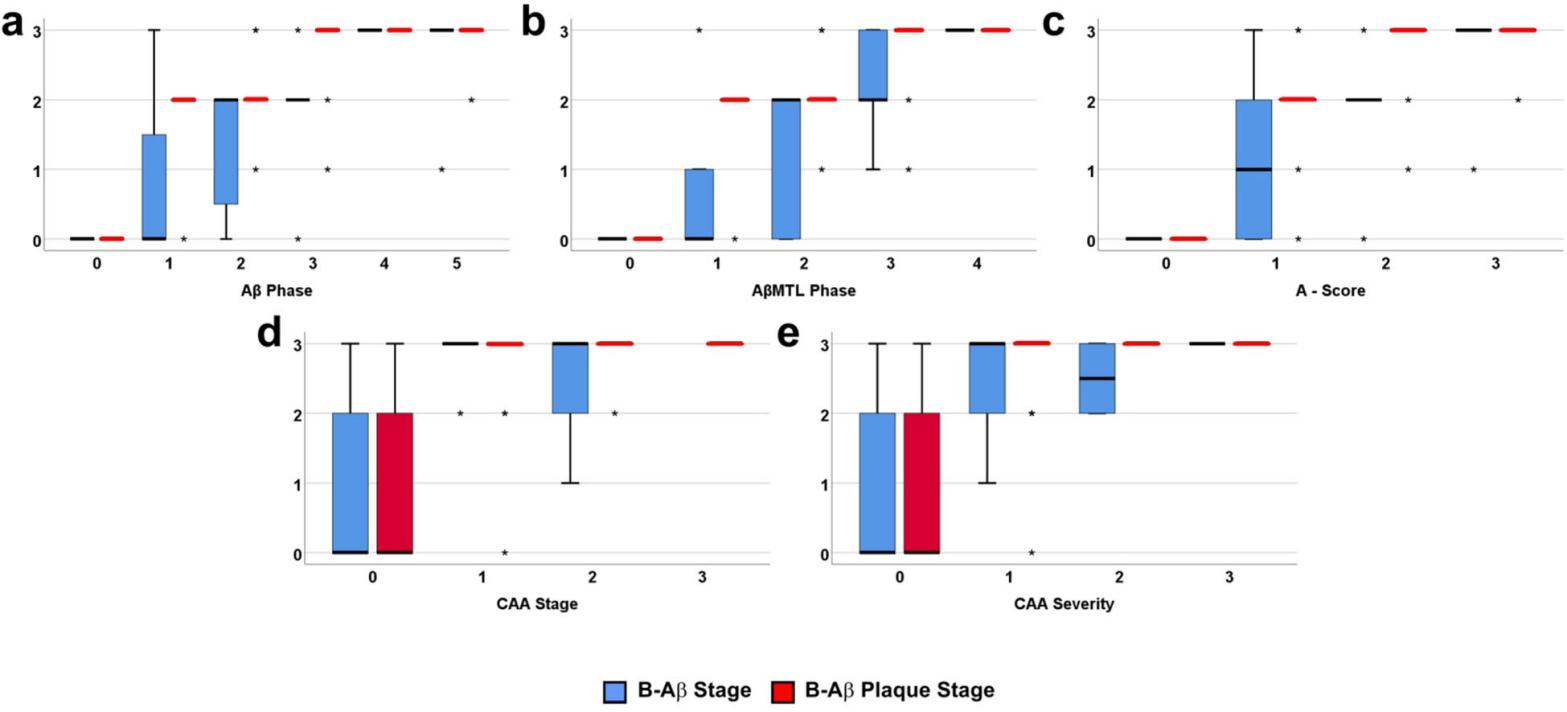
Boxplot diagram comparing the relationship of the Aβ phases (**a**), AβMTL phases (**b**), A-scores (**c**), CAA stages (**d**) and CAA severities (**e**) with the biochemical stages of Aβ aggregate maturation reflecting the hierarchical occurrence of Aβ, Aβ_N3pE_, and Aβ_pSer8_ in Aβ aggregates in brain homogenates (B-Aβ stages) or in Aβ plaques (B-Aβ plaque stage) in cohort 1. **a**: The B-Aβ stages and the B-Aβ plaque stages increased with increasing Aβ phase until Aβ phase 4 when the maximum B-Aβ and B-Aβ plaques stages were reached. **b, c**: AβMTL phases and A-scores also correlated with the B-Aβ stages and B-Aβ plaque stages but exhibiting no ceiling effect. **d, e**: In the presence of CAA regardless of the stage (**d**) or severity (**e**), end-stage B-Aβ stage and B-Aβ plaque stage were observed, probably indicative for a ceiling effect. (Spearman correlation analysis: *r* = 0.694–0.906) (for detailed statistical analysis see Additional file 1: Table S5a)

The qualitative changes in the composition of Aβ aggregates over time as represented by the B-Aβ stages and B-Aβ plaques stages progressed with increasing Aβ phase, AβMTL phase and A-score (Fig. 3a-c). With respect to the Aβ phases the last stage of Aβ aggregate maturation was reached in nearly all Aβ phase 4 cases and remained stable in Aβ phase 5 (Fig. 3a). Such a saturation effect was not that apparent when studying the B-Aβ and B-Aβ plaque stages in relation to the AβMTL phases and the A-scores (Fig. 3b, c). Most cases with CAA (CAA stage 1–3, CAA severity degree 1–3) showed B-Aβ stage 3 (66.7%) and B-Aβ plaque stage 3 (87.1%) (Fig. 3d, e).

### Correlations of PET-Aβ phase estimates with topographical parameters of Aβ pathology and Aβ load

In the cases from cohort 3 we compared the different topographical parameters for Aβ pathology (Aβ phase, AβMTL phase, A-score, CAA stage and CAA severity) as well as the quantitative measure of the Aβ load among the PET-Aβ phase estimates obtained in patients 0–846 (mean 215) days before death and subsequent autopsy. All topographical parameters of Aβ pathology as well as the Aβ load correlated with the PET-Aβ phase estimates with r ranging from 0.610 to 0.835 (Spearman correlation analysis: *p* < 0.001) (Additional file 1: Table S6).

**Fig. 4:**
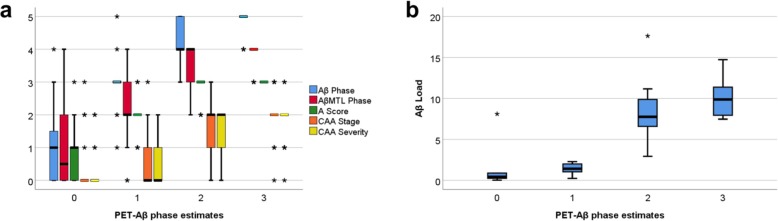
Boxplot diagrams representing the distribution of Aβ phase, AβMTL phase, A-score, CAA stage, CAA severity (**a**) and Aβ loads (**b**) in relation to the PET-Aβ phase estimate in cohort 3. All parameters correlated with the PET-Aβ phase estimates (*r* = 0.610–0.835; *p* < 0.001; for detailed statistical analysis see Additional file 1: Table S6). Note that the main increase in Aβ load occurs after Aβ became detectable in PET-Aβ phase estimate 1. The mean value of the Aβ load in PET-Aβ phase estimate 1 was 1.396% (median 1.42%) indicative for the threshold of Aβ detectability in the brain by PET

Of the 20 amyloid PET-negative cases (PET-Aβ phase estimate 0), 13 showed plaque pathology whereas CAA was found only in four of them. With increasing PET-Aβ phase estimate the Aβ phases increased (Fig. 4a). AβMTL phase, A-score, CAA stage and CAA severity also gradually increased until PET-Aβ phase estimate 2 reaching a plateau that is also seen in cases with PET-Aβ phase estimate 3. Of note, CAA stage remained stable at the second stage while the third CAA stage was limited to single cases with PET-Aβ phase estimates ranging from 0 to 3. For CAA severity only one case with PET-Aβ phase estimate 3 exhibited CAA-related bleedings eligible for severity degree 3 (Fig. 4a). The Aβ load also gradually increased with increasing PET-Aβ phase estimate becoming detectable with a median Aβ load of 1.42% in PET-Aβ phase estimate 1 (Fig. 4b).

### Correlations of topographical, quantitative and qualitative Aβ parameters with the degree of dementia and non-Aβ AD pathology

**Fig. 5:**
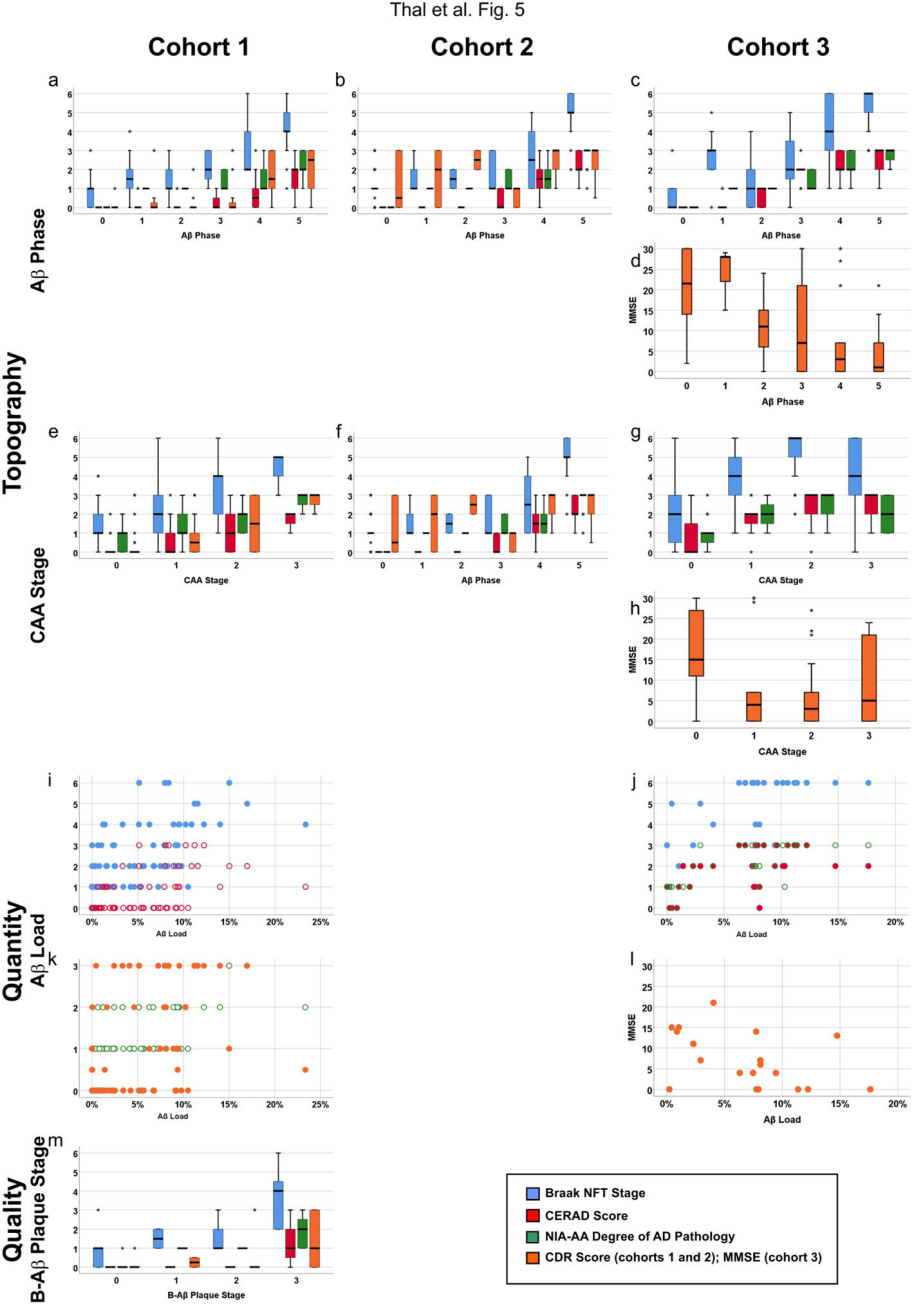
Boxplot and scatter diagrams depicting the correlation of the Braak NFT stages, CERAD-scores for neuritic plaque pathology, NIA-AA scores of AD pathology, and the clinical dementia scores (CDR for cohorts 1 and 2 and MMSE for cohort 3) with the topographical Aβ parameters Aβ phase (**a-d**) and CAA stage (**e-h**), the quantitative measure of the Aβ load (**i-l**), and the qualitative aspect provided by the B-Aβ plaque stages (**m**). The boxplots are depicted separately for cohorts 1 (**a, e, i, k, m**), 2 (**b, f**), and 3 (**c, d, g, h, j, l**). The Braak NFT stages, CERAD scores, NIA-AA degrees of AD pathology, and CDR scores correlated with all parameters depicted here (*r* = 0.287–0.920, *p* < 0.001). Likewise, the MMSE scores showed a negative correlation with the Aβ phase and the CAA stages in cohort 3 (*r* = − 0.514/− 0.315, *p* ≤ 0.012) except for the Aβ load (*p* = 0.051) which showed only a trend (for detailed statistical analysis see Additional file 1: Table S7)

To determine the relationship of the different aspects of Aβ pathology with NFT pathology, neuritic plaques, AD pathology in general and the degree of dementia, we performed partial correlation analysis controlled for age and sex. The topographical and qualitative Aβ parameters as well as the Aβ, Aβ_N3pE_, and Aβ_pSer8_ load correlated with Braak NFT stages, CERAD scores, NIA-AA degrees of AD pathology, and the degree of dementia measured by the CDR score or the MMSE score, respectively (Fig. 5a-l, Additional file 1: Table S7a-c). The biochemically measured levels of soluble, dispersible, membrane-associated, and plaque associated Aβ, Aβ_N3pE_, and plaque-associated Aβ_pSer8_ also correlated with increasing Braak NFT stages, CERAD scores, and NIA-AA degrees of AD. However, only soluble, dispersible, plaque-associated Aβ_N3pE_, and plaque-associated Aβ_pSer8_ correlated with the CDR score whereas the biochemical levels of non-modified forms of Aβ did not correlate with increasing dementia represented by the CDR score (Fig. 5, Additional file 1: Table S7). The B-Aβ stage and the B-Aβ plaque stages representing qualitative changes in Aβ aggregates/ plaque composition over time correlated with Braak NFT stages, CERAD scores, NIA-AA degrees of AD pathology and CDR-scores indicating that the presence of post-translationally modified forms of Aβ is associated with cognitive decline and AD pathology progression (Fig. 5m, Additional file 1: Table S7a).

A few cases did not follow the general correlation between Aβ and NFT pathology. Sixteen cases showed widespread amyloid plaque pathology with Aβ phases 4 and 5 but only low Braak NFT stages (0-II). Another 4 cases exhibited severe NFT pathology (Braak NFT stage IV-V) with negligible plaque pathology (Aβ phases 0–2). Moreover, severe CAA without large numbers of plaques or NFTs was seen in one case of cohort 3 (Braak NFT stage 0, amyloid phase 3, CAA stage 3). Altogether, 21 of 271 cases (7.75%) did not follow the general correlation.

The PET-Aβ phase estimates as an Aβ topography-related parameter that can be measured in patients correlated with Braak NFT stage, CERAD scores and NIA-AA degrees of AD pathology and with decreasing MMSE scores indicating a correlation with cognitive decline (Additional file 1: Table S7c). All cases with PET-Aβ phase estimates between 2 and 3 with known MMSE scores showed at least mild cognitive impairment (MMSE score 27 or lower) with a median of 3 (mean = 5.19) and a range between 0 and 27. Except for one case with vascular dementia (NIA-AA score 1, Braak NFT stage 3, Aβ phase 3) all other cases (39 out of 40 cases) fulfilled the criteria of intermediate to severe AD pathology (NIA-AA scores 2 and 3). PET-Aβ phase estimate 1 cases consisted of a heterogeneous group with MMSE scores ranging between 0 and 30 (median: 7; mean = 9.71). The NIA-AA degree of AD pathology was low (1) in 70% of the PET-Aβ phase 1 cases and intermediate (2) in 30%. Those cases with NIA-AA scores of 2 were demented due to AD whereas the cases with NIA-AA scores of 1 were either normal or had dementia due to Lewy body disease or vascular dementia. The neuropathologically normal case with low NIA-AA degree of AD pathology and cognitive deficits was reported to be terminally ill (scan-death interval: 9 days), which may explain the low performance in the MMSE test. The 20 cases with a negative [18F]flutemetamol PET were either normal (*n* = 7) or had a non-AD dementia (Lewy body disease (*n* = 3), vascular dementia (*n* = 6), neurofibrillary predominant dementia (*n* = 1), argyrophilic grain disease (*n* = 1), progressive supranuclear palsy (*n* = 1), and frontotemporal lobar degeneration with TDP-43 pathology (FTLD-TDP) (*n* = 1)). The MMSE scores (available in 15 of these cases) ranged between 0 and 30 with a median of 15 (mean = 15.15). AD pathology was either absent or low, except for the one case with FTLD-TDP with an intermediate degree of AD pathology.

## Discussion

The results of this study demonstrate that topographical, quantitative and qualitative aspects of Aβ pathology correlated with one another. The advancing Aβ pathology detected neuropathologically in autopsy brains correlated well with the increase of [^18^F]flutemetamol PET-derived PET-Aβ phase estimates, i.e. with a staging system based upon amyloid PET-derived SUVR-thresholds applicable in patients during life [67]. All different aspects of Aβ pathology also correlated with increasing non-Aβ AD neuropathology, i.e. Braak NFT stages and CERAD scores for neuritic plaques detected with an antibody raised against p-τ. The NIA-AA score of AD pathology as a global parameter for AD pathology linking Aβ and p-τ lesions and the degree of dementia correlated with increasing topographical, quantitative, and qualitative aspects of Aβ pathology. Biochemically, the levels of post-translationally modified forms of Aβ pathology, i.e. Aβ_N3pE_ and Aβ_pSer8_, correlated with the increasing degree of dementia as represented by the CDR score but not with the levels of non-modified Aβ. This argues in favor of the hypothesis that qualitative changes in Aβ aggregate composition, i.e. Aβ aggregate maturation due to posttranslational modifications of Aβ, are critical in the progression of AD. This maturation of Aβ aggregates in plaques also correlated with the frequency of neuritic plaques (Additional file 1: Table S7a), i.e. the development of amyloid plaques associated with p-τ-containing dystrophic neurites. These findings are in line with previous reports showing a stepwise progression of Aβ aggregate maturation in AD, CAA, and Down-syndrome [4, 24, 44, 57] as well as with studies indicating the aggregation prone effects of Aβ_N3pE_ and Aβ_pSer8_ [55, 63].

Our results are in line a) with previous studies showing correlations between Aβ plaque loads, qualitative changes in Aβ aggregate composition and the topographical distribution of Aβ plaque pathology [3, 57, 75], b) with the association of the respective Aβ parameters with NFT and neuritic plaque pathology and c) with the degree of dementia when including non-AD control, preclinical and symptomatic AD cases [11, 12, 54, 69, 71–73]. Although the degree of dementia has been reported to correlate better with NFT pathology in AD cases rather than with Aβ plaque pathology [2], it became clear that Aβ pathology has already reached high levels when AD becomes symptomatic while NFT pathology did not [30, 71, 73]. The correlation of AD progression with increasing tracer retention in amyloid PET seen in our study is in line with other studies [15, 17, 31, 52, 60].

In contrast to previous studies, we correlated all aspects (topography, quantity and quality) of Aβ pathology in one cohort, confirmed the relationship between topographical aspects of Aβ pathology with p-τ pathology in NFTs, neuritic plaques and the cognitive status of the patients in two additional cohorts. Based on our findings the assessment of the Aβ phases already provides sufficient information to estimate the quantity of Aβ plaques, their maturation status, the frequency of neuritic plaques, the severity of CAA, and the topographical expansion of NFTs in more than 90% of the cases. Accordingly, the PET-Aβ phase estimates as an in-vivo parameter represent distinct steps of the underlying neuropathological and biochemical progression of Aβ pathology. Since changes in Aβ biomarkers, including those observed with amyloid PET, precede that of p-τ biomarkers [13, 30] and since PET-Aβ phase estimates allow prediction of the underlying neuropathological phase of Aβ deposition [67] our current findings strongly argue in favor of using amyloid PET and its derived PET-Aβ phase estimates as markers for AD pathology in general and for disease monitoring once the diagnosis of AD has been established in a given individual.

Although our results demonstrate strong correlations among the AD-related neuropathological and biochemical parameters studied here, there are some exceptional cases with discrepancies among the aspects of Aβ and AD pathology: Severe CAA without large numbers of plaques or NFTs was seen in one case of cohort 3 (Braak NFT stage 0, amyloid phase 3, CAA stage 3); widespread amyloid plaques (Aβ phases 4 and 5) but only limited NFT pathology (Braak NFT stage 0-II) was observed in 16 cases; and severe NFT pathology (Braak NFT stage IV-V) with negligible plaque pathology (Aβ phases 0–2) occurred in 4 cases [7, 32]. Moreover, AD-related pathology changes are frequently accompanied by changes related to vascular incidents [5, 27, 77] or other types of neurodegenerations, such as Lewy body pathology or other tauopathies [6, 32, 37, 74, 78]. Therefore, amyloid PET and its power for estimating AD pathology alone is, in our opinion, not sufficient for establishing the diagnosis of AD and needs to be supplemented by a neurological examination, magnetic resonance imaging to screen for vascular lesions, and p-τ biomarkers in order to confirm the diagnosis of AD, to identify specific variants such as the plaque predominant form of AD, and to detect non-AD tauopathies as it is part of the current recommendations for the clinical diagnosis of AD [49]. This is documented by cases in cohort 3 with advanced Aβ pathology with high PET-Aβ phase estimates and additional non-AD pathology (e.g. Lewy body disease). For disease progression monitoring on the other hand, PET-Aβ phase estimates of a single patient at different points in time could be a powerful tool to assess the speed of disease progression because of its close association with the neuropathological markers, especially the Aβ phase [67]. The transition from preclinical AD to the symptomatic stage correlated with a transition from Aβ phase 3 to 4 [71]. Since these Aβ phases can be assessed by the PET-Aβ phase estimates, amyloid PET can indicate patients at high risk for this transition. In advanced symptomatic AD, p-τ may be a better progression marker [30].

Another finding of this study is the correlation between the aspects of Aβ pathology with CAA, and the correlation of CAA severity and the topographical distribution of CAA with the PET-Aβ phase estimate. These data are in line with previous findings on the correlation between CAA and Aβ plaque deposition [68], the detectability of CAA cases by amyloid PET [14, 39] and its probable characteristics in the cortical tracer retention pattern [8, 19, 22]. However, it was also reported that CAA had no major impact on amyloid PET positivity because of the correlation between Aβ plaques and CAA [36]. Only single cases with predominant CAA and very limited amounts of plaques became detectable by amyloid PET as reported by others [20] and confirmed by one CAA-stage 3 case in our cohort 3 exhibiting Aβ phase 3 and Braak NFT-stage 0. This indicates that amyloid PET did not distinguish between Aβ plaque pathology and vascular Aβ deposition in CAA with the algorithms currently applied in a routine diagnostic setting. It just indicates whether Aβ deposits in a given amount are present or not whereby in most cases plaque pathology and CAA anyway correlate with one another. However, our findings also show single amyloid PET negative cases with CAA. Accordingly, we cannot confirm that amyloid PET is capable of ruling out CAA completely when negative as suggested by other authors [8] although indeed 16/20 amyloid PET negative cases in our study had no CAA. It may be important to note that one case with end-stage CAA (CAA distributed all over the brain) in our sample was amyloid PET negative. When assessing only positive/negative amyloid PET reads it had been reported that CAA may contribute to amyloid positivity in phase 3 cases [36] whereas analysis of the SUVRs allowed distinction between amyloid phases 0–2, 3, 4, and 5 in the same cohort of cases without interference with CAA [67].

Many reports showed that τ pathology precedes Aβ plaques neuropathologically [12, 16, 21, 65, 73]. In our three cohorts, we confirmed the presence of early stages of τ pathology in cases without Aβ plaques (Fig. 1) known as primary age-related tauopathy (PART) and to precede AD pathology [21]. Accordingly, our findings do not support the amyloid hypothesis in the sense that Aβ causes τ pathology [64]. However, the parallel increase of p-τ and Aβ pathology in the autopsy brains supports the hypothesis that Aβ can drive the propagation of pre-existing p-τ pathology as recently demonstrated in amyloid precursor protein transgenic mice [26, 28, 45, 53]. As such, our findings are in line with the hypothesis that prevalent τ pathology fulfilling the criteria of definite PART (= presence of NFTs in the absence of Aβ plaques) is a prerequisite for the development of AD and can be accelerated by the presence of Aβ aggregates finally leading to AD [65].

A limitation of this study is that there is no assessment of the B-Aβ stages and B-Aβ plaques stages in cohort 3 for comparison with the [^18^F]flutemetamol PET images. Since cohort 3 was recruited and studied in the context of a phase III clinical trial to determine the diagnostic value of [^18^F]flutemetamol, the assessment of Aβ_N3pE_ presence or Aβ_pSer8_ presence was not considered in the study protocol. Correlation of the [^18^F]flutemetamol PET derived PET-Aβ phase estimates with the AβMTL phases and the Aβ load, i.e. a second parameter describing the topographical distribution of Aβ plaques (AβMTL phase) in addition to the Aβ phases and a quantitative parameter (Aβ load), support the hypothesis that the relationship between the three aspects of Aβ pathology as shown in cohort 1 is also valid for other cohorts of cases.

A second limitation of this study is the fact that we do not have an independent control group to confirm that the PET-Aβ phase estimates indeed correlate with the underlying Aβ phase. In addition to statistical analysis using bootstrapping methods as previously published [67], we determined a second parameter representing the topographical distribution of Aβ plaque pathology, the AβMTL phase, which is based on the assessment of a different set of brain regions in comparison to the Aβ phases [71, 72] and which also correlated very well with the PET-Aβ phase estimates. Moreover, another group of researchers demonstrated a similar amyloid PET-based distinction between the Aβ phases when using PIB-PET-derived centiloids to compare their cases [42]. [^18^F]flutemetamol shows, thereby, similar amyloid detection properties when compared with Pittsburg compound B (PIB) in clinical studies [33, 43] and similar increases in tracer retention with increasing Aβ phase [42, 67].

## Conclusions

Our analysis of Aβ pathology in its topographical, quantitative and qualitative aspects (incl. CAA), in its relationship with other histopathological features of AD, cognitive decline and in its detection by amyloid PET in patients during life showed that all these parameters are correlated with one another. In doing so, the determination of one of these parameters seems to be sufficient for estimating the others, and for monitoring the progression of a once diagnosed symptomatic or preclinical disease. However, the occurrence of few cases (7.75% in our three cohorts) that lie outside of the general correlation, e.g., cases with plaque-predominant AD, argues against the use of only one parameter for establishing the diagnosis of AD. Since amyloid PET is the clinical biomarker, which is best validated by post-mortem end-of-life studies [15, 36, 42, 60, 67], and which allows estimation of the underlying neuropathological phase of Aβ deposition [67], amyloid PET seems to be ideally suited for this purpose especially for studying and determining the critical transition from preclinical to symptomatic AD as indicated by its correlation with the Aβ phases, Aβ loads, Braak NFT stages and the CERAD scores for neuritic plaque pathology.

## Supplementary information

**Additional file 1: Table S1.** List of antibodies and silver techniques. IHC = immunohistochemistry, WB = western blotting. **Table S2.** Assessment of topographical distribution of Aβ plaques (a-d), CAA (a, e), and CAA severity [80] (a, f). Tissue-block selection (a) and assessment of Aβ phases (b) [71], AβMTL phases (c) [72], A-scores (d) [35], CAA stages (e), and the CAA severity degree according to Vonsattel et al. [80] (f). **Table S3.** Assessment of the biochemical Aβ stage of plaque maturation (=B-Aβ plaque stage; a) and the biochemical stage of Aβ aggregate maturation in brain lysates (B-Aβ stage; b). **Table S4.** Assessment of PET-Aβ phase estimates as previously published [67]. **Table S5.** Spearman correlation analysis between Aβ phases, AβMTL phases, A-scores, and Aβ load as assessed in cohorts 1 (a), 2 (b), and 3 (c) as well as Aβ_N3pE_ load, Aβ_pSer8_ load, B-Aβ stage, B-Aβ plaque stage, and the levels of soluble, dispersible, membrane-associated and plaque-associated Aβ, Aβ_N3pE_, and Aβ_pSer8_ in cohort 1. r and *p*-values are provided. No adjustment for age and sex because different methods assessing Aβ pathology were compared. n = number of cases compared. **Table S6.** Spearman correlation analysis between PET-Aβ phase estimates, topographical and quantitative Aβ parameters assessed in cohort 3. No adjustment for age and sex because different methods assessing Aβ pathology were compared. n = number of cases compared. **Table S7.** Partial correlation analysis controlled for age and sex between NFT stages, CERAD scores of neuritic plaque pathology, NIA-AA degree of AD pathology, CDR-scores, Aβ phases, AβMTL phases, A-scores, and Aβ load as assessed in cohorts 1 (a), 2 (b), and 3 (c) as well as Aβ_N3pE_ load, Aβ_pSer8_ load, B-Aβ stage, B-Aβ plaque stage, and the levels of soluble, dispersible, membrane-associated and plaque-associated Aβ, Aβ_N3pE_, and Aβ_pSer8_ in cohort 1. r and p-values are provided. n = number of cases compared.

## Data Availability

The anonymized datasets used and/or analyzed during the current study are stored in UZ/KU-Leuven network drives and available from the corresponding author on reasonable request.
