# Correction to: Different aspects of Alzheimer’s disease-related amyloid β-peptide pathology and their relationship to amyloid positron emission tomography imaging and dementia

**DOI:** 10.1186/s40478-020-01005-5

**Published:** 2020-08-03

**Authors:** Dietmar Rudolf Thal, Alicja Ronisz, Thomas Tousseyn, Ajeet Rijal Upadhaya, Karthikeyan Balakrishnan, Rik Vandenberghe, Mathieu Vandenbulcke, Christine A. F. von Arnim, Markus Otto, Thomas G. Beach, Johan Lilja, Kerstin Heurling, Aruna Chakrabarty, Azzam Ismail, Christopher Buckley, Adrian P. L. Smith, Sathish Kumar, Gill Farrar, Jochen Walter

**Affiliations:** 1 Department of Imaging and Pathology, KU-Leuven, Leuven, Belgium; 2 Department of Pathology, UZ-Leuven, Leuven, Belgium; 3 Leuven Brain Institute, KU-Leuven, Leuven, Belgium; 4 Laboratory for Neuropathology – Institute of Pathology, University of Ulm, Ulm, Germany; 5 Department of Gene Therapy, University of Ulm, Ulm, Germany; 6 Department of Neurosciences, KU-Leuven, Herestraat 49, 3000 Leuven, Belgium; 7 Department of Neurology, UZ-Leuven, Leuven, Belgium; 8 Department of Geriatric Psychiatry, UZ-Leuven, Leuven, Belgium; 9 Department of Neurology, University of Ulm, Ulm, Germany; 10 Department of Geriatrics, University Medical Center Göttingen, Göttingen, Germany; 11 Civin Laboratory for Neuropathology, Banner Sun Health Research Institute, Sun City, AZ USA; 12 Hermes Medical Solutions AB, Stockholm, Sweden; 13 Department of Psychiatry and Neurochemistry, Wallenberg Centre for Molecular and Translational Medicine, University of Gothenburg, Gothenborg, Sweden; 14 Pathology and Tumour Biology, Leeds Institute of Molecular Medicine, St. James Hospital, Leeds, UK; 15 GE Healthcare Life Sciences, Amersham, UK; 16 Department of Neurology, University of Bonn, Bonn, Germany

**Correction to: Acta Neuropathol Commun 7, 178 (2019)**

**https://doi.org/10.1186/s40478-019-0837-9**

In the publication of the original article [1], Fig. 5f had an incorrect diagram.

The original figure shows in panel F the diagram: Aβ Phase

The correct diagram for panel F is: CAA-stage

Aβ Phase was already provided in Fig. 5b

The updated Fig. 5 is published in this correction article.

**Fig. 5:**
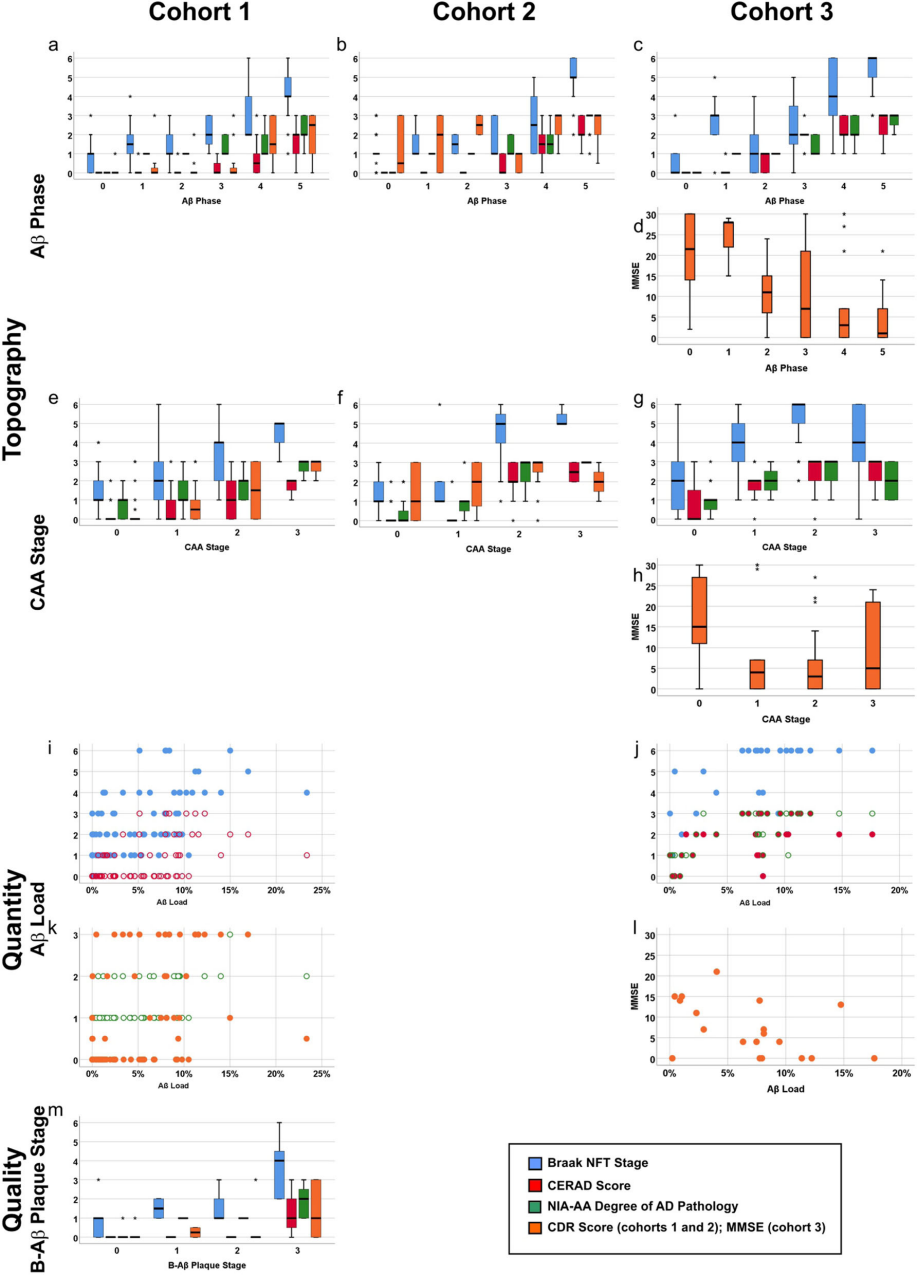
Boxplot and scatter diagrams depicting the correlation of the Braak NFT stages, CERAD-scores for neuritic plaque pathology, NIA-AA scores of AD pathology, and the clinical dementia scores (CDR for cohorts 1 and 2 and MMSE for cohort 3) with the topographical Aβ parameters Aβ phase (**a-d**) and CAA stage (**e-h**), the quantitative measure of the Aβ load (**i-l**), and the qualitative aspect provided by the B-Aβ plaque stages (**m**). The boxplots are depicted separately for cohorts 1 (**a, e, i, k, m**), 2 (**b, f**), and 3 (**c, d, g, h, j, l**). The Braak NFT stages, CERAD scores, NIA-AA degrees of AD pathology, and CDR scores correlated with all parameters depicted here (*r* = 0.287–0.920, *p* < 0.001). Likewise, the MMSE scores showed a negative correlation with the Aβ phase and the CAA stages in cohort 3 (*r* = − 0.514/− 0.315, *p* ≤ 0.012) except for the Aβ load (*p* = 0.051) which showed only a trend (for detailed statistical analysis see Additional file 1: Table S7)

## References

[1] Thal DR, Ronisz A, Tousseyn T (2019). Different aspects of Alzheimer’s disease-related amyloid β-peptide pathology and their relationship to amyloid positron emission tomography imaging and dementia. Acta Neuropathol Commun.

