# Supplementary material for: Different aspects of Alzheimer’s disease-related amyloid β-peptide pathology and their relationship to amyloid positron emission tomography imaging and dementia
Source: Acta Neuropathol Commun. 2019 Nov 14;7:178. doi: 10.1186/s40478-019-0837-9 (PMC6854805; doi:10.1186/s40478-019-0837-9)
Supplement: Supplementary file 1 — Additional file 1: Table S1. List of antibodies and silver techniques. IHC = immunohistochemistry, WB = western blotting. Table S2. Assessment of topographical distribution of Aβ plaques (a-d), CAA (a, e), and CAA severity [80] (a, f). Tissue-block selection (a) and assessment of Aβ phases (b) [71], AβMTL phases (c) [72], A-scores (d) [35], CAA stages (e), and the CAA severity degree according to Vonsattel et al. [80] (f). Table S3. Assessment of the biochemical Aβ stage of plaque maturation (=B-Aβ plaque stage; a) and the biochemical stage of Aβ aggregate maturation in brain lysates (B-Aβ stage; b). Table S4. Assessment of PET-Aβ phase estimates as previously published [67]. Table S5. Spearman correlation analysis between Aβ phases, AβMTL phases, A-scores, and Aβ load as assessed in cohorts 1 (a), 2 (b), and 3 (c) as well as AβN3pE load, AβpSer8 load, B-Aβ stage, B-Aβ plaque stage, and the levels of soluble, dispersible, membrane-associated and plaque-associated Aβ, AβN3pE, and AβpSer8 in cohort 1. r and p-values are provided. No adjustment for age and sex because different methods assessing Aβ pathology were compared. n = number of cases compared. Table S6. Spearman correlation analysis between PET-Aβ phase estimates, topographical and quantitative Aβ parameters assessed in cohort 3. No adjustment for age and sex because different methods assessing Aβ pathology were compared. n = number of cases compared. Table S7. Partial correlation analysis controlled for age and sex between NFT stages, CERAD scores of neuritic plaque pathology, NIA-AA degree of AD pathology, CDR-scores, Aβ phases, AβMTL phases, A-scores, and Aβ load as assessed in cohorts 1 (a), 2 (b), and 3 (c) as well as AβN3pE load, AβpSer8 load, B-Aβ stage, B-Aβ plaque stage, and the levels of soluble, dispersible, membrane-associated and plaque-associated Aβ, AβN3pE, and AβpSer8 in cohort 1. r and p-values are provided. n = number of cases compared. [file 40478_2019_837_MOESM1_ESM.docx]

**Additional file 1: Table S1 – S7**

**Table S1: List of antibodies and silver techniques.**

**IHC = immunohistochemistry, WB = western blotting.**

| **Antibody** | **Antigen** | **Host** | **Clone** | **Concentration IHC** | **Concentration WB** | **Pretreatment for IHC** | **Source** | **Specificity in WB** | **Purpose of Use** | **Applied in Cohort** |
| --- | --- | --- | --- | --- | --- | --- | --- | --- | --- | --- |
| anti-  Aβ_17-24_ | Aβ 17-24 | mouse | 4G8 | 1/5000 |  | formic acid | Covance, USA |  | Determination of Aβ phases, AβMTL phase, B-Aβ plaque stages, Aβ load, and CAA. This antibody detects full-length as well as N-terminal truncated forms of non-modified and modified Aβ­_40/42_ [1]. | 1-3 |
| anti-  Aβ_1-17_ | Aβ 1-17 | mouse | 6E10 |  | 1/1000 | n.a. | Covance, USA | 5 ng/ml [2] | Determination of biochemical Aβ levels and B-Aβ stage. This antibody detects full-length forms of Aβ­_40/42_ but does not react with Aβ_pSer8_ [1]. | 1 |
| anti-Aβ_N3pE_ | Aβ N3pE | rabbit | polyclonal | 1/100 | 1/500 | formic acid, heat pretreatment at pH6 | IBL, Japan | ~20 ng/ml [3] | Determination of B-Aβ stage, B-Aβ plaque stages, biochemical levels of Aβ_N3pE_ and Aβ_N3pE_ load | 1 |
| anti-Aβ_pSer8_ | Aβ pSer8 | mouse | 1E4E11 | 1/5 | 1/50 | formic acid, heat pretreatment at pH6 | (Kumar et al., 2013) gift Prof. Walter, Bonn, Germany | 25 ng/ml [4] | Determination of B-Aβ stage, B-Aβ plaque stages, biochemical levels of Aβ_pSer8_ and Aβ_pSer8_ load | 1 |
| anti-abnormal τ protein | phosphorylated τ (pSer 202 and pThr205) | mouse | AT8 | 1/1000 |  | - | Thermo-Scientific, USA |  | Assessment of Braak NFT stages, neuritic plaques (CERAD scores were based on these stainings) and accompanying τ pathology | 1-3 |
| anti-pTDP43 | phosphorylated TDP43 (pSer 409/410) | rabbit | polyclonal | 1/5000 |  | heat pretreatment at pH6 | Cosmobio, Japan |  | Assessment of AD-related and non-AD related pTDP43 pathology for neuropathological diagnosis | 1, 2 |
| anti-α-synuclein | α-synuclein | mouse | 5G4 | 1/2000 |  | formic acid, heat pretreatment at pH6 | Millipore, USA |  | Assessment of α-synuclein pathology for neuropathological diagnosis | 1, 2 |
| anti-α-synuclein | α-synuclein | mouse | NCL-L-ASYN | 1/40 |  | heat pretreatment at pH6 | Leica, UK |  | Assessment of α-synuclein pathology for neuropathological diagnosis | 3 |
| anti-ubiquitin | ubiquitin | rabbit | polyclonal | 1/400 |  | heat pretreatment at pH6 | DakoCytomation, Denmark |  | Assessment of AD-related and non-AD related ubiquitin pathology for neuropathological diagnosis | 3 |

| **Silver methods** | **Protocol reference** | **Purpose of Use** | **Applied in Cohort** |
| --- | --- | --- | --- |
| Gallyas staining | Braak, H., and E. Braak. 1991. 'Demonstration of amyloid deposits and neurofibrillary changes in whole brain sections', Brain Pathol, 1: 213-6. [5] | Confirmation of Braak NFT staging and assessment of neuritic plaques | 1 |
| Bielschowsky staining | Uchihara, T. 2007. 'Silver diagnosis in neuropathology: principles, practice and revised interpretation', Acta Neuropathol, 113: 483-99. [6] | Assessment of neuritic plaques | 3 |

**Table S2:** Assessment of topographical distribution of Aβ plaques (a-d), CAA (a, e), and CAA severity [7] **(a, f).** a: Tissue-block selection (**a**) and assessing Aβ phases (**b**)[8], AβMTL phases (**c**)[9], A-scores (**d**)[10], CAA stages (**e**) , and the CAA severity degree according to Vonsattel et al. [7] (**f**).

**a** *Required brain samples to be stained with the 4G8 antibody against Aβ:*

|  | **Aβ phases** | **AβMTL phases** | **A-score** | **CAA-stage** | **CAA severity** |
| --- | --- | --- | --- | --- | --- |
| 1. Anterior hippocampus with entorhinal cortex | + | + | + | + | + |
| 2. Posterior hippocampus at the level of the lateral geniculate body | + | + | + | + | + |
| 3. Occipital cortex with primary visual cortex | + |  | + | + | + |
| 4. Basal ganglia | + |  | + | + |  |
| 5. Midbrain | + |  | + | + |  |
| 6. Cerebellum | + |  | + | + |  |

**b** *Assessment of Aβ phases* *[8]:*

The following brain regions are scores separately for the presence/absence of Aβ plaque pathology.

This protocol is based on the assessment of anti-Aβ_17-24_ or anti-Aβ_42_ stained plaques.

The numbers in brackets indicate the sample block in which a given region is assessed

| **Aβ plaques** | **present** | **absent** |
| --- | --- | --- |
| a. Temporal neocortex (1, 2) |  |  |
| b. Occipital neocortex (3) |  |  |
| c. Entorhinal cortex (1) |  |  |
| d. Hippocampus/subiculum/presubiculum (1, 2) |  |  |
| e. Amygdala (if present in 1) |  |  |
| f. Caudate nucleus (4) |  |  |
| g. Putamen (4) |  |  |
| h. Substantia nigra (5) |  |  |
| i. Colliculi superiors or inferiors (5) |  |  |
| j. Central (periaqueductal) gray matter (5) |  |  |
| k. Red nucleus (5) |  |  |
| l. Raphe nuclei/reticular formation (5) |  |  |
| m. Cerebellar cortex (6) |  |  |

**Aβ phase**: 0 = (no plaques present)

1 = (Plaques present in a or b)

2 = (Plaques present in a, b and c, d, or e)

3 = (Plaques present in a-e and f or g)

4 = (Plaques present in a-g and h, i, j, k, or l)

5 = (Plaques present in a-g and h, i, j, k, or l and m)

**c** *Assessment of AβMTL phases [9]:*

The following brain regions are scores separately for the presence/absence of Aβ plaque pathology.

This protocol is based on the assessment of anti-Aβ_17-24_ or anti-Aβ_42_ stained plaques.

The numbers in brackets indicate the sample block in which a given region is assessed

| **Aβ plaques** | **present** | **absent** |
| --- | --- | --- |
| a. Temporal neocortex (1, 2) |  |  |
| b. Occipital neocortex (3) |  |  |
| c. Entorhinal cortex layers pre-β to pri-γ (1) |  |  |
| d. Entorhinal cortex layer pre-α (1) |  |  |
| e. Subiculum (1, 2) |  |  |
| f. Hippocampus sector CA1 (1, 2) |  |  |
| g. Outer molecular layer of the dentate gyrus (1, 2) |  |  |
| h. Hippocampus sector CA4 (1,2) |  |  |

**AβMTL phase:** 0 = (no plaques present)

1 = (Plaques present in a or b)

2 = (Plaques present in a, b and c, e, or f)

3 = (Plaques present in a-c, f and g)

4 = (Plaques present in a-c, f, g and d or h)

**d** *Assessment of A-score [10]:*

**A-score:** 0 = Aβ phase 0

1 = Aβ phase 1 or 2

2 = Aβ phase 3

3 = Aβ phase 4 or 5

**e** *Assessment of CAA stages [11].*

The following brain regions are scores separately for the presence/absence of vascular Aβ deposits.

This protocol is based on the assessment of anti-Aβ_17-24_ or anti-Aβ_42_ stained CAA pathology.

The numbers in brackets indicate the sample block in which a given region is assessed

| **CAA** | **present** | **absent** |
| --- | --- | --- |
| a. Temporal neocortex + leptomeninges (1, 2) |  |  |
| b. Occipital neocortex + leptomeninges (3) |  |  |
| c. Entorhinal cortex + leptomeninges (1) |  |  |
| d. Hippocampus/subiculum/presubiculum (1, 2) |  |  |
| e. Amygdala (if present in 1) |  |  |
| f. Caudate nucleus (4) |  |  |
| g. Putamen (4) |  |  |
| h. Midbrain (5) |  |  |
| i. Pons |  |  |
| j. Cerebellar cortex (6) |  |  |

**CAA stage**: 0 = (no CAA present)

1 = (CAA present in a or b)

2 = (CAA present in a, b and c, d, e, or j)

3 = (CAA present in a-e,j and f, g, h, or i)

**f** *Assessment of CAA severity [7].*

The brain regions indicated in a are screened for the gradual affection of the vessel wall by CAA.

| **CAA-severity pattern** | **present** | **absent** |
| --- | --- | --- |
| a. focal Aβ deposits in the vessel wall without significant destruction of the lamina media |  |  |
| b. The lamina media is replaced by Aβ deposits |  |  |
| c. CAA-associated bleedings or microbleeds |  |  |

**CAA severity**: 0 = (no CAA present)

1 = (CAA-affected vessels exhibiting the severity pattern of a)

2 = (CAA-affected vessels exhibiting the severity pattern of a and b)

3 = (CAA-affected vessels exhibiting the severity pattern of a-c)

**Table S3**: Assessment of the biochemical Aβ stage of plaque maturation (=B-Aβ plaque stage; a) and the biochemical stage of Aβ aggregate maturation in brain lysates (B-Aβ stage; b).

**a** B-Aβ plaque stage:

| 0 | = | no plaques; |
| --- | --- | --- |
| 1 | = | plaques stained only with antibodies detecting non-modified forms of Aβ (4G8); but negative for anti-Aβ_N3pE_ and anti-Aβ_pSer8_ antibodies. |
| 2 | = | plaques stained with antibodies detecting non-modified forms of Aβ (4G8) and anti-Aβ_N3pE_; but negative for anti-Aβ_pSer8_ antibodies |
| 3 | = | plaques stained with antibodies detecting non-modified forms of Aβ (4G8), anti-Aβ_N3pE_, and anti-Aβ_pSer8_ [12]. |

**b** B-Aβ stage

| 0 | = | no Aβ |
| --- | --- | --- |
| 1 | = | Aβ detected only with antibodies detecting non-modified forms of Aβ (anti-Aβ_1-17_); but negative for anti-Aβ_N3pE_ and anti-Aβ_pSer8_ antibodies. |
| 2 | = | Aβ detected with antibodies detecting non-modified forms of Aβ (anti-Aβ_1-17_) and anti-Aβ_N3pE_; but negative for anti-Aβ_pSer8_ antibodies. |
| 3 | = | Aβ detected with antibodies detecting non-modified forms of Aβ (anti-Aβ_1-17_), and with anti-Aβ_N3pE_, and anti-Aβ_pSer8_ antibodies[12]. |

**Table S4:** Assessment of PET-Aβ phase estimates as previously published [13]

| PET-Aβ phase estimate 0 | = | Aβ phases 0-1(2) | = | SUVRneo < 0.5 and/or SUVRcaud < 0.6; |
| --- | --- | --- | --- | --- |
| PET-Aβ phase estimate 1 | = | Aβ phases 2-3 | = | SUVRneo ≥ 0.5 - < 0.6 and/or SUVRcaud ≥ 0.6 - < 0.7; |
| PET-Aβ phase estimate 2 | = | Aβ phase 4 | = | SUVRneo ≥ 0.6 and/or SUVRcaud ≥ 0.7 - ≤1.0; |
| PET-Aβ phase estimate 3 | = | Aβ phase 5 | = | SUVRcaud > 1.0. |

**Table S5**: Spearman correlation analysis between Aβ phases, AβMTL phases, A-scores, and Aβ load as assessed in cohorts 1 (**a**), 2 (**b**), and 3 (**c**) as well as Aβ_N3pE_ load, Aβ_pSer8_ load, B-Aβ stage, B-Aβ plaque stage, and the levels of soluble, dispersible, membrane-associated and plaque-associated Aβ, Aβ_N3pE_, and Aβ_pSer8_ in cohort 1. r and p-values are provided. No adjustment for age and sex because different methods assessing Aβ pathology were compared. n = number of cases compared.

**a**: Cohort 1

| **Cohort 1** | **Aβ phase** | **AβMTL phase** | **A-score** | **CAA Stage** | **CAA Severity** | **Aβ load** | **B-Aβ stage** | **B-Aβ plaque stage** | **n** |
| --- | --- | --- | --- | --- | --- | --- | --- | --- | --- |
| AβMTL phase | r = 0.965; p < 0.001 | n.d. |  |  |  |  |  |  | 95 |
| A-score | r = 0.978; p < 0.001 | r = 0.941; p < 0.001 | n.d. |  |  |  |  |  | 95 |
| CAA stage | r = 0.739, p < 0.001 | r = 0.695, p < 0.001 | r = 0.728, p < 0.001 | n.d. |  |  |  |  | 95 |
| CAA severity | r = 0.703, p < 0.001 | r = 0.663, p < 0.001 | r = 0.704, p < 0.001 | r = 0.962, p < 0.001 | n.d. |  |  |  | 95 |
| Aβ load | r = 0.888, p < 0.001 | r = 0.899, p < 0.001 | r = 0.868, p < 0.001 | r = 0.630, p < 0.001 | r = 0.582, p < 0.001 | n.d. |  |  | 95 |
| Aβ_N3pE_ load | r = 0.882, p < 0.001 | r = 0.872, p < 0.001 | r = 0.865, p < 0.001 | r = 0.731, p < 0.001 | r = 0.721, p < 0.001 | r = 0.932, p < 0.001 | r = 0.765, p < 0.001* | r = 0.860, p < 0.001 | 70, *38 |
| Aβ_pSer8_ load | r = 0.810, p < 0.001 | r = 0.835, p < 0.001 | r = 0.809, p < 0.001 | r = 0.777, p = 0.001 | r = 0.806, p < 0.001 | r = 0.756, p < 0.001 | r = 0.836, p < 0.001* | r = 0.813, p < 0.001 | 70, *38 |
| B-Aβ stage | r = 0.821, p < 0.001 | r = 0.866, p < 0.001 | r = 0.832, p < 0.001 | r = 0.694, p < 0.001 | r = 0.709, p = 0.002 | r = 0.815, p < 0.001 | n.d. | r = 0.757, p < 0.001 | 38 |
| B-Aβ plaque stage | r = 0.896, p < 0.001 | r = 0.906, p < 0.001 | r = 0.905, p < 0.001 | r = 0.730, p < 0.001 | r = 0.736, p < 0.001 | r = 0.867, p < 0.001 |  | n.d. | 70 |
| Soluble Aβ | r = 0.683, p < 0.001 | r = 0.789, p < 0.001 | r = 0.668, p < 0.001 | r = 0.619, p < 0.001 | r = 0.603, p < 0.001 | r = 0.715, p < 0.001 | r = 0.793, p < 0.001 | r = 0.699, p < 0.001 | 38 |
| Dispersible Aβ | r = 0.735, p < 0.001 | r = 0.832, p < 0.001 | r = 0.709, p < 0.001 | r = 0.696, p < 0.001 | r = 0.719, p < 0.001 | r = 0.728, p < 0.001 | r = 0.839, p < 0.001 | r = 0.685, p < 0.001 | 38 |
| Membrane-associated Aβ | r = 0.730, p < 0.001 | r = 0.809, p < 0.001 | r = 0.725, p < 0.001 | r = 0.629, p < 0.001 | r = 0.628, p < 0.001 | r = 0.758, p < 0.001 | r = 0.776, p < 0.001 | r = 0.781, p < 0.001 | 38 |
| Plaque-associated Aβ | r = 0.667, p < 0.001 | r = 0.749, p < 0.001 | r = 0.664, p < 0.001 | r = 0.514, p = 0.001 | r = 0.519, p = 0.001 | r = 0.725, p < 0.001 | r = 0.690, p < 0.001 | r = 0.770, p < 0.001 | 38 |
| Soluble Aβ_N3pE_ | r = 0.637, p < 0.001 | r = 0.692, p < 0.001 | r = 0.640, p < 0.001 | r = 0.598, p < 0.001 | r = 0.627, p < 0.001 | r = 0.572, p < 0.001 | r = 0.728, p < 0.001 | r = 0.503, p = 0.001 | 38 |
| Dispersible Aβ_N3pE_ | r = 0.625, p < 0.001 | r = 0.669, p < 0.001 | r = 0.619, p < 0.001 | r = 0.594, p < 0.001 | r = 0.636, p < 0.001 | r = 0.613, p < 0.001 | r = 0.726, p < 0.001 | r = 0.503, p = 0.001 | 38 |
| Membrane-associated Aβ_N3pE_ | r = 0.679, p < 0.001 | r = 0.756, p < 0.001 | r = 0.698, p < 0.001 | r = 0.707, p < 0.001 | r = 0.723, p < 0.001 | r = 0.725, p < 0.001 | r = 0.888, p < 0.001 | r = 0.730, p < 0.001 | 38 |
| Plaque-associated Aβ_N3pE_ | r = 0.666, p < 0.001 | r = 0.701, p < 0.001 | r = 0.657, p < 0.001 | r = 0.591, p < 0.001 | r = 0.613, p < 0.001 | r = 0.589, p < 0.001 | r = 0.725, p < 0.001 | r = 0.499, p = 0.001 | 38 |
| Soluble Aβ_pSer8_ | n.d. | n.d. | n.d. | n.d. | n.d. | n.d. | n.d. | n.d. | 38 |
| Dispersible Aβ_pSer8_ | r = 0.524, p = 0.001 | r = 0.556, p < 0.001 | r = 0.497, p = 0.002 | r = 0.521, p = 0.001 | r = 0.521, p = 0.001 | r = 0.477, p = 0.002 | r = 0.543, p < 0.001 | r = 0.406, p = 0.011 | 38 |
| Membrane-associated Aβ_pSer8_ | r = 0.367, p = 0.024 | r = 0.413, p = 0.010 | r = 0.370, p = 0.022 | r = 0.364, p = 0.025 | r = 0.465, p = 0.003 | r = 0.323, p = 0.048 | r = 0.431, p = 0.007 | r = 0.323, p = 0.048 | 38 |
| Plaque-associated Aβ_pSer8_ | r = 0.324, p = 0.047 | r = 0.340, p = 0.037 | r = 0.373, p = 0.021 | r = 0.348, p = 0.032 | r = 0.415, p = 0.010 | r = 0.170, p = 0.308 | r = 0.369, p = 0.023 | p = 0.094 | 38 |

**b**: Cohort 2

| **Cohort 2** | **Aβ phase** | **AβMTL phase** | **A-score** | **CAA Stage** | **CAA Severity** | **Aβ load** | **B-Aβ stage** | **B-Aβ plaque stage** | **n** |
| --- | --- | --- | --- | --- | --- | --- | --- | --- | --- |
| AβMTL phase | r = 0.983; p < 0.001 | n.d. |  |  |  |  |  |  | 79 |
| A-score | r = 0.990; p < 0.001 | r = 0.976; p < 0.001 | n.d. |  |  |  |  |  | 79 |
| CAA stage | r = 0.758, p < 0.001 | r = 0.718, p < 0.001 | r = 0.736, p < 0.001 | n.d. |  |  |  |  | 79 |
| CAA severity | r = 0.664, p < 0.001 | r = 0.622, p < 0.001 | r = 0.642, p < 0.001 | r = 0.944, p < 0.001 | n.d. |  |  |  | 79 |

**c**: Cohort 3

| **Cohort 3** | **Aβ phase** | **AβMTL phase** | **A-score** | **CAA Stage** | **CAA Severity** | **Aβ load** | **B-Aβ stage** | **B-Aβ plaque stage** | **n** |
| --- | --- | --- | --- | --- | --- | --- | --- | --- | --- |
| AβMTL phase | r = 0.835; p < 0.001 | n.d. |  |  |  |  |  |  | 97 |
| A-score | r = 0.887; p < 0.001 | r = 0.841; p < 0.001 | n.d. |  |  |  |  |  | 97 |
| CAA stage | r = 0.664, p < 0.001 | r = 0.575, p < 0.001 | r = 0.623, p < 0.001 | n.d. |  |  |  |  | 97 |
| CAA severity | r = 0.637, p < 0.001 | r = 0.626, p < 0.001 | r = 0.644, p < 0.001 | r = 0.823, p < 0.001 | n.d. |  |  |  | 97 |
| Aβ load | r = 0.701, p < 0.001 | r = 0.760, p < 0.001 | r = 0.680, p < 0.001 | r = 0.667, p < 0.001 | r = 0.707, p < 0.001 | n.d. |  |  | 31 |

**Table S6:** Spearman correlation analysis between PET-Aβ phase estimates, topographical and quantitative Aβ parameters assessed in cohort 3. No adjustment for age and sex because different methods assessing Aβ pathology were compared. n = number of cases compared.

| **Cohort 3** | **Aβ phase** | **AβMTL phase** | **A-score** | **CAA Stage** | **CAA Severity** | **Aβ load** | **n** |
| --- | --- | --- | --- | --- | --- | --- | --- |
| PET-Aβ phase estimate | r = 0.835; p < 0.001 | r = 0.759; p < 0.001 | r = 0.814; p < 0.001 | r = 0.610; p < 0.001 | r = 0.669; p < 0.001 | r = 0.712; p < 0.001* | 97/*31 |

**Table S7:** Partial correlation analysis controlled for age and sex between NFT stages, CERAD scores of neuritic plaque pathology, NIA-AA degree of AD pathology, CDR-scores, Aβ phases, AβMTL phases, A-scores, and Aβ load as assessed in cohorts 1 (a), 2 (b), and 3 (c) as well as Aβ_N3pE_ load, Aβ_pSer8_ load, B-Aβ stage, B-Aβ plaque stage, and the levels of soluble, dispersible, membrane-associated and plaque-associated Aβ, Aβ_N3pE_, and Aβ_pSer8_ in cohort 1. r and p-values are provided. n = number of cases compared.

**a: Cohort 1**

| **Cohort 1** | **NFT Stage** | **CERAD** | **NIA-AA** | **CDR** | **n** |
| --- | --- | --- | --- | --- | --- |
| Aβ phase | r = 0.614; p < 0.001 | r = 0.596; p < 0.001 | r = 0.786; p < 0.001 | r = 0.449; p < 0.001* | 95/*88 |
| AβMTL phase | r = 0.606; p < 0.001 | r = 0.572; p < 0.001 | r = 0.793; p < 0.001 | r = 0.369; p < 0.001* | 95/*88 |
| A-score | r = 0.618; p < 0.001 | r = 0.575; p < 0.001 | r = 0.782; p < 0.001 | r = 0.450; p < 0.001* | 95/*88 |
| CAA stage | r = 0.402, p < 0.001 | r = 0.466; p < 0.001 | r = 0.528; p < 0.001 | r = 0.431; p < 0.001* | 95/*88 |
| CAA severity | r = 0.352, p < 0.001 | r = 0.412, p < 0.001 | r = 0.483, p < 0.001 | r = 0.420, p < 0.001* | 95/*88 |
| Aβ load | r = 0.489, p < 0.001 | r = 0.441, p < 0.001 | r = 0.554, p < 0.001 | r = 0.295, p < 0.001* | 95/*88 |
| Aβ_N3pE_ load | r = 0.670, p < 0.001 | r = 0.626, p < 0.001 | r = 0.687, p < 0.001 | r = 0.386, p = 0.002* | 70, *63 |
| Aβ_pSer8_ load | r = 0.289, p = 0.017 | r = 0.484, p < 0.001 | r = 0.267, p = 0.028 | r = 0.441, p < 0.001* | 70, *63 |
| B-Aβ stage | r = 0.603, p < 0.001 | r = 0.576, p < 0.001 | r = 0.640, p < 0.001 | r = 0.287, p = 0.025* | 38, *32 |
| B-Aβ plaque stage | r = 0.549, p < 0.001 | r = 0.427, p < 0.001 | r = 0.726, p < 0.001 | r = 0.287, p < 0.001* | 70, *63 |
| Soluble Aβ | r = 0.532, p = 0.001 | r = 0.536, p = 0.001 | r = 0.556, p < 0.001 | p = 0.377* | 38, *32 |
| Dispersible Aβ | r = 0.568, p < 0.001 | r = 0.630, p < 0.001 | r = 0.627, p < 0.001 | p = 0.338* | 38, *32 |
| Membrane-associated Aβ | r = 0.578, p < 0.001 | r = 0.620, p < 0.001 | r = 0.635, p < 0.001 | p = 0.186* | 38, *32 |
| Plaque-associated Aβ | r = 0.472, p = 0.004 | r = 0.510, p = 0.001 | r = 0.572, p < 0.001 | p = 0.261* | 38, *32 |
| Soluble Aβ_N3pE_ | r = 0.736, p < 0.001 | r = 0.756, p < 0.001 | r = 0.759, p < 0.001 | r = 0.371, p = 0.044* | 38, *32 |
| Dispersible Aβ_N3pE_ | r = 0.624, p < 0.001 | r = 0.694, p < 0.001 | r = 0.672, p < 0.001 | r = 0.429, p = 0.018* | 38, *32 |
| Membrane-associated Aβ_N3pE_ | r = 0.488, p = 0.003 | r = 0.491, p = 0.002 | r = 0.526, p = 0.001 | p = 0.689* | 38, *32 |
| Plaque-associated Aβ_N3pE_ | r = 0.772, p < 0.001 | r = 0.785, p < 0.001 | r = 0.781, p < 0.001 | r = 0.371, p = 0.043* | 38, *32 |
| Soluble Aβ_pSer8_ | n.d. | n.d. | n.d. | n.d. | 38, *32 |
| Dispersible Aβ_pSer8_ | p = 0.070 | p = 0.092 | r = 0.335, p = 0.045 | p = 0.411* | 38, *32 |
| Membrane-associated Aβ_pSer8_ | p = 0.154 | r = 0.334, p = 0.047 | p = 0.162 | p = 0.528* | 38, *32 |
| Plaque-associated Aβ_pSer8_ | r = 0.602, p < 0.001 | r = 0.618, p < 0.001 | r = 0.533, p = 0.001 | r = 0.420, p = 0.021* | 38, *32 |

**b**: Cohort 2

| **Cohort 2** | **NFT Stage** | **CERAD** | **NIA-AA** | **CDR** | **n** |
| --- | --- | --- | --- | --- | --- |
| Aβ phase | r = 0.762; p < 0.001 | r = 0.855; p < 0.001 | r = 0.920; p < 0.001 | r = 0.341; p = 0.003* | 97,*74 |
| AβMTL phase | r = 0.710; p < 0.001 | r = 0.803; p < 0.001 | r = 0.896; p < 0.001 | r = 0.290; p = 0.013* | 97,*74 |
| A-score | r = 0.710; p < 0.001 | r = 0.823; p < 0.001 | r = 0.902; p < 0.001 | r = 0.330; p = 0.005* | 97,*74 |
| CAA stage | r = 0.730, p < 0.001 | r = 0.760; p < 0.001 | r = 0.800; p < 0.001 | r = 0.314; p = 0.007* | 97,*74 |
| CAA severity | r = 0.538, p < 0.001 | r = 0.546; p < 0.001 | r = 0.625; p < 0.001 | r = 0.265, p = 0.024* | 97,*74 |

**c**: Cohort 3

| **Cohort 3** | **NFT Stage** | **CERAD** | **NIA-AA** | **MMSE** | **n** |
| --- | --- | --- | --- | --- | --- |
| Aβ phase | r = 0.700; p < 0.001 | r = 0.748; p < 0.001 | r = 0.835; p < 0.001 | r = -0.514; p < 0.001* | 97,*65 |
| AβMTL phase | r = 0.683; p < 0.001 | r = 0.752; p < 0.001 | r = 0.816; p < 0.001 | r = -0.506; p < 0.001* | 97,*65 |
| A-score | r = 0.668; p < 0.001 | r = 0.761; p < 0.001 | r = 0.812; p < 0.001 | r = -0.476; p < 0.001* | 97,*65 |
| CAA stage | r = 0.592, p < 0.001 | r = 0.528; p < 0.001 | r = 0.609; p < 0.001 | r = -0.315; p = 0.012* | 97,*65 |
| CAA severity | r = 0.566, p < 0.001 | r = 0.512, p < 0.001 | r = 0.607, p < 0.001 | r = -0.368, p = 0.003* | 97,*65 |
| Aβ load | r = 0.609, p < 0.001 | r = 0.579, p = 0.001 | r = 0.705, p < 0.001 | p = 0.051* | 31, *19 |
| PET-Aβ phase estimate | r = 0.704; p < 0.001 | r = 0.708; p < 0.001 | r = 0.817; p < 0.001 | r = -0.467; p < 0.001* | 97,*65 |

**References**

1. Kumar S, Rezaei-Ghaleh N, Terwel D, Thal DR, Richard M, Hoch M, Mc Donald JM, Wullner U, Glebov K, Heneka MT, Walsh DM, Zweckstetter M, Walter J (2011) Extracellular phosphorylation of the amyloid beta-peptide promotes formation of toxic aggregates during the pathogenesis of Alzheimer's disease. EMBO J 30 (11):2255-2265. doi:emboj2011138 [pii] 10.1038/emboj.2011.138

2. Youmans KL, Tai LM, Kanekiyo T, Stine WB, Jr., Michon SC, Nwabuisi-Heath E, Manelli AM, Fu Y, Riordan S, Eimer WA, Binder L, Bu G, Yu C, Hartley DM, LaDu MJ (2012) Intraneuronal Abeta detection in 5xFAD mice by a new Abeta-specific antibody. Mol Neurodegener 7:8. doi:1750-1326-7-8 [pii]

10.1186/1750-1326-7-8

3. Saido TC, Iwatsubo T, Mann DM, Shimada H, Ihara Y, Kawashima S (1995) Dominant and differential deposition of distinct beta-amyloid peptide species, A beta N3(pE), in senile plaques. Neuron 14 (2):457-466

4. Kumar S, Wirths O, Theil S, Gerth J, Bayer TA, Walter J (2013) Early intraneuronal accumulation and increased aggregation of phosphorylated Abeta in a mouse model of Alzheimer's disease. Acta Neuropathol 125 (5):699-709. doi:10.1007/s00401-013-1107-8

5. Braak H, Braak E (1991) Demonstration of amyloid deposits and neurofibrillary changes in whole brain sections. Brain Pathol 1 (3):213-216.

6. Uchihara T (2007) Silver diagnosis in neuropathology: principles, practice and revised interpretation. Acta Neuropathol 113 (5):483-499. doi:10.1007/s00401-007-0200-2

7. Vonsattel JP, Myers RH, Hedley-Whyte ET, Ropper AH, Bird ED, Richardson EP, Jr. (1991) Cerebral amyloid angiopathy without and with cerebral hemorrhages: a comparative histological study. Ann Neurol 30 (5):637-649. doi:10.1002/ana.410300503

8. Thal DR, Rüb U, Orantes M, Braak H (2002) Phases of Abeta-deposition in the human brain and its relevance for the development of AD. Neurology 58:1791-1800

9. Thal DR, Rüb U, Schultz C, Sassin I, Ghebremedhin E, Del Tredici K, Braak E, Braak H (2000) Sequence of Abeta-protein deposition in the human medial temporal lobe. J Neuropathol Exp Neurol 59 (8):733-748.

10. Hyman BT, Phelps CH, Beach TG, Bigio EH, Cairns NJ, Carrillo MC, Dickson DW, Duyckaerts C, Frosch MP, Masliah E, Mirra SS, Nelson PT, Schneider JA, Thal DR, Thies B, Trojanowski JQ, Vinters HV, Montine TJ (2012) National Institute on Aging–Alzheimer’s Association guidelines for the neuropathologic assessment of Alzheimer’s disease. Alzheimers Dement 8:1-13

11. Thal DR, Ghebremedhin E, Orantes M, Wiestler OD (2003) Vascular pathology in Alzheimer’s disease: Correlation of cerebral amyloid angiopathy and arteriosclerosis / lipohyalinosis with cognitive decline. J Neuropathol Exp Neurol 62 (12):1287-1301

12. Rijal Upadhaya A, Kosterin I, Kumar S, Von Arnim C, Yamaguchi H, Fändrich M, Walter J, Thal DR (2014) Biochemical stages of amyloid β-peptide aggregation and accumulation in the human brain and their association with symptomatic and pathologically-preclinical Alzheimer's disease. Brain 137:887-903

13. Thal DR, Beach TG, Zanette M, Lilja J, Heurling K, Chakrabarty A, Ismail A, Farrar G, Buckley C, Smith APL (2018) Estimation of amyloid distribution by [(18)F]flutemetamol PET predicts the neuropathological phase of amyloid beta-protein deposition. Acta Neuropathol 136 (4):557-567. doi:10.1007/s00401-018-1897-9
